# Supplementary material for: Elongation factor-specific capture of RNA polymerase II complexes
Source: Cell Rep Methods. 2022 Dec 9;2(12):100368. doi: 10.1016/j.crmeth.2022.100368 (PMC9795356; doi:10.1016/j.crmeth.2022.100368)
Supplement: Document S1. Figures S1–S5 and Methods S1 [file mmc1.pdf]

Cell Reports Methods, Volume *2*

Supplemental information

Elongation factor-specific capture

of RNA polymerase II complexes

Lea H. Gregersen, Richard Mitter, and Jesper Q. Svejstrup

Figure S1. Establishment of ELCAP, related to Figure 1

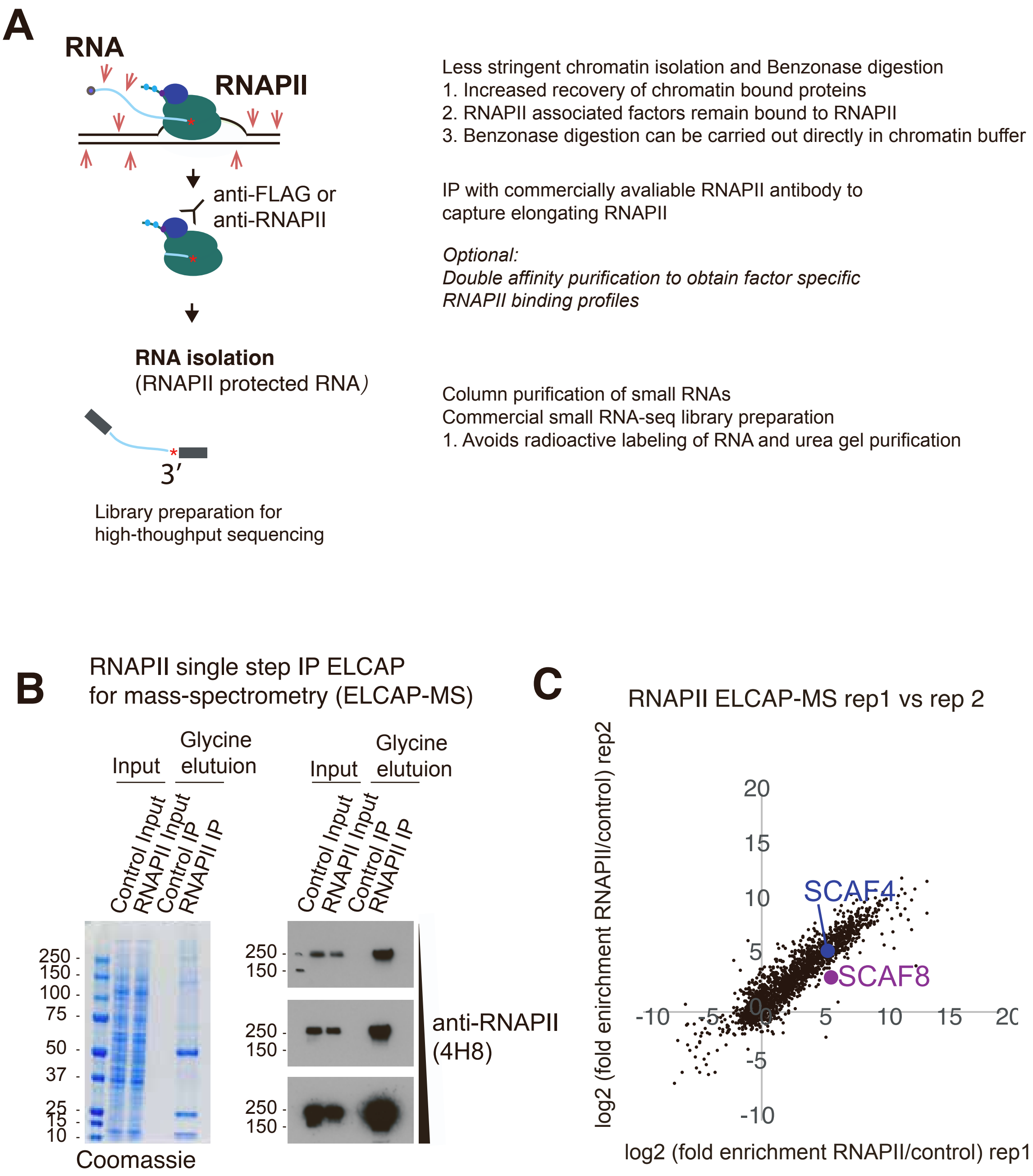

Figure S2. Comparison of RNAPII ELCAP-MS and RNAPII mNET-MS, related to Figure 2 and 3

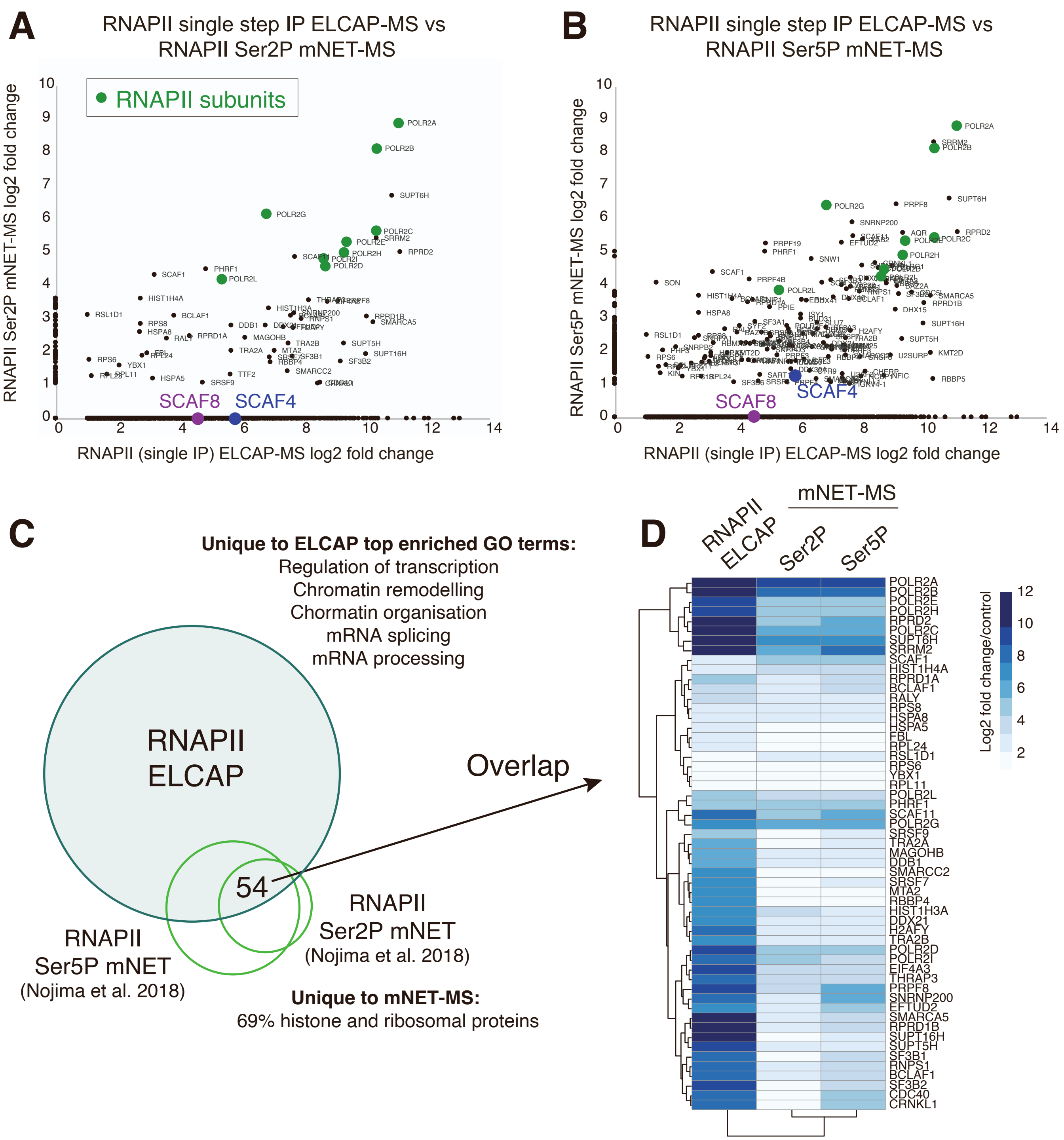

Figure S3. Single and double ELCAP purification of RNAPII, SCAF4- and SCAF8-RNAPII, related to Figure 4 and 5

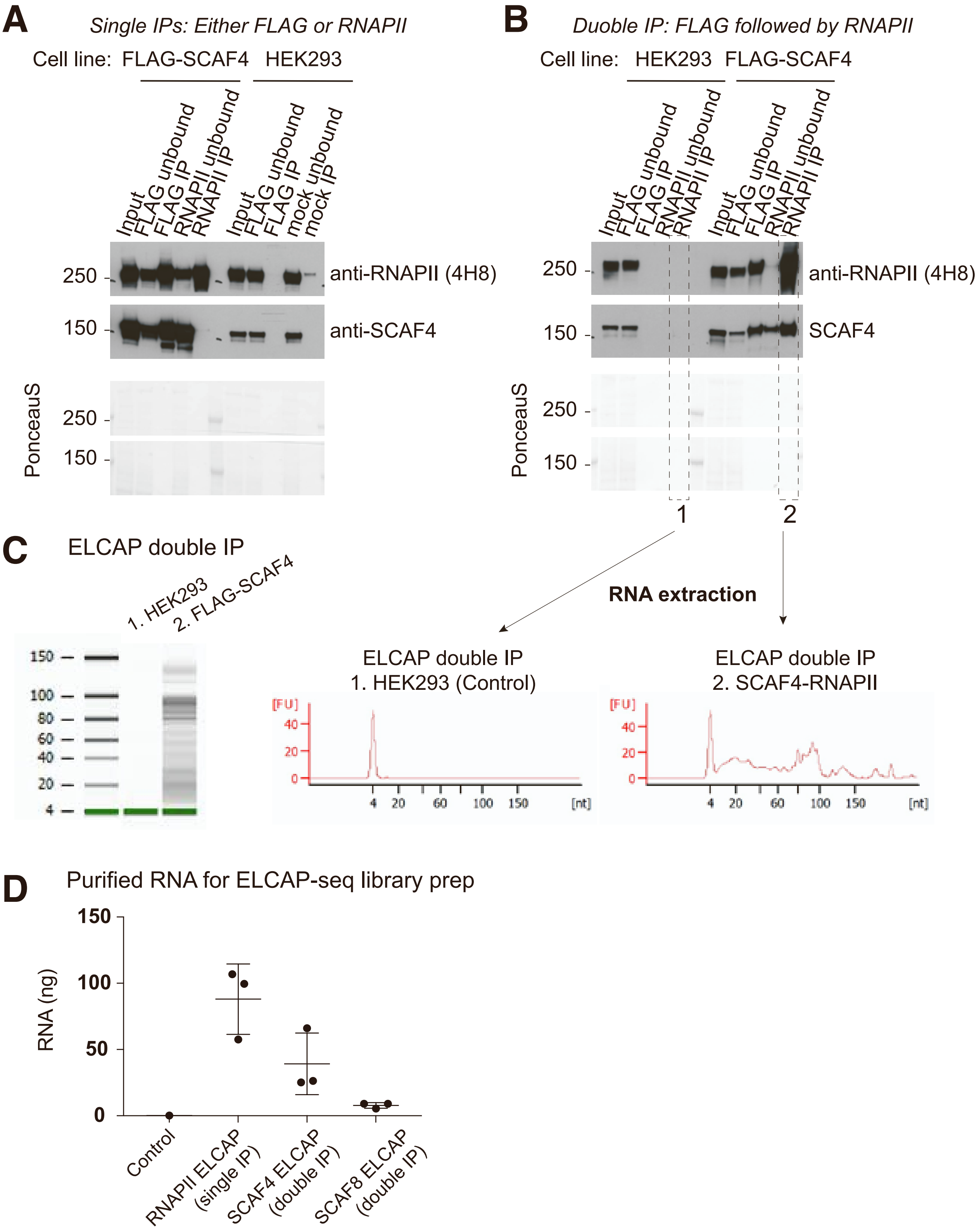

Figure S4. RNAPII ELCAP-seq profiles compared to ChIP-seq data, related to Figure 4

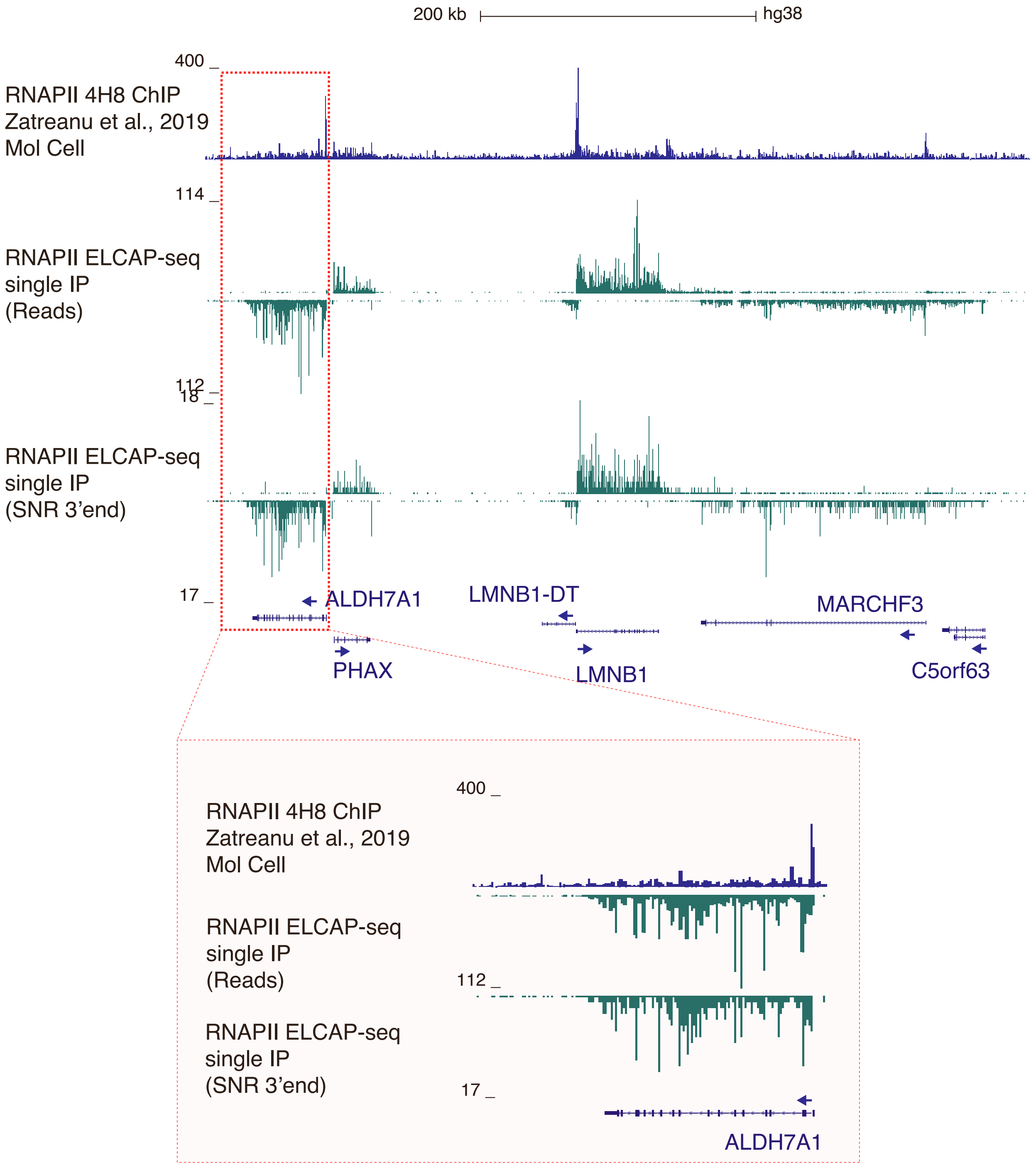

Figure S5. Transcriptionally engaged RNAPII, SCAF4-RNAPII and SCAF8-RNAPII ELCAP-seq profiles, related to Figure 5 and 6

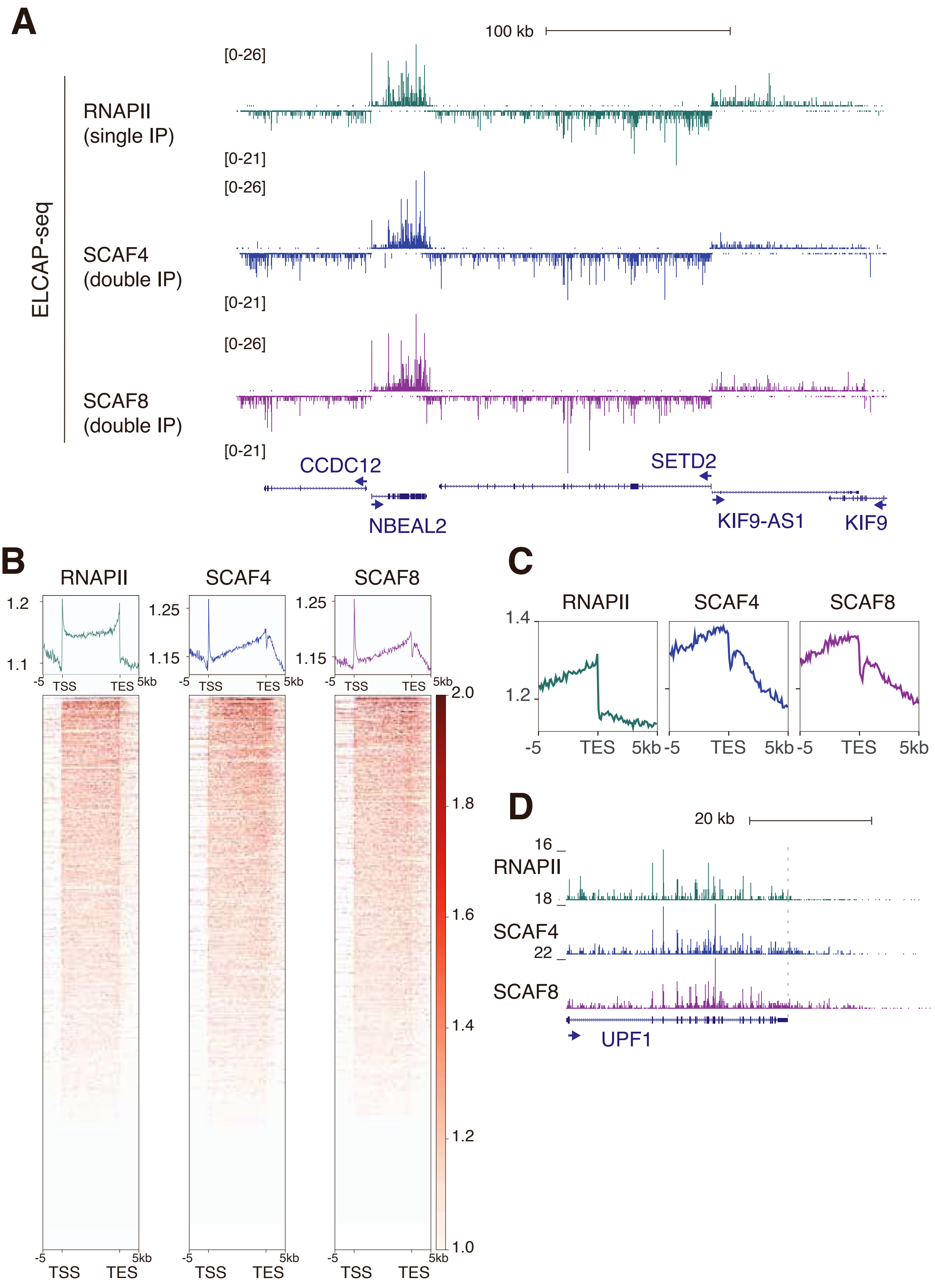

Supplemental Figure Legends

Supplemental Fig. 1. Establishment of ELCAP.

(**A**) Outline of single immunoprecipitation step of either RNAPII or FLAG-SCAF4. (**B**) Coomassie stain and Western blot of a single-step IP RNAPII purification for proteomics. Mock (IgG) IP was performed as a negative control (**C**) Correlation of log2 fold change enrichment between the two RNAPII ELCAP-MS (single IP) biological replicates.

Supplemental Fig. 2. Comparison of RNAPII ELCAP-MS and RNAPII mNET-MS.

(**A**) RNAPII ELCAP-MS (single step IP) log2 fold changes compared to RNAPII mNET-seq mass-spectrometry results for RNAPII complexes immunoprecipitated using a RNAPII CTD Ser2P antibody[1]. (**B**) As in A, but compared to RNAPII mNET-MS results from immunoprecipitation using a RNAPII CTD Ser5P antibody. (**C**) Venn diagram of RNAPII interactors identified with a log2 fold change >2 using RNAPII ELCAP-MS, RNAPII Ser2P mNET-MS and RNAPII Ser5P mNET-seq. (**D**) Heatmap showing 54 proteins with a log2 FC >2 using RNAPII ELCAP-MS, RNAPII Ser2P mNET-MS and RNAPII Ser5P mNET-seq. mNET-seq MS data is previously desribed[1].

Supplemental Fig. 3. Single and double ELCAP purification of RNAPII, SCAF4- and SCAF8-RNAPII.

(**A**) Single IP of FLAG-SCAF4 or RNAPII from stable FLAG-SCAF4 expressing cell lines. A FLAG IP or mock (IgG coupled to dynabeads) from HEK293 cells were used as negative controls (right part of gels). (**B**) Double affinity purification of SCAF4-RNAPII carried out by an initial FLAG IP, FLAG peptide elution and a subsequent RNAPII IP. A double affinity purification from HEK293 cells not expressing any FLAG-tag was used as a negative control (left side of gels). (**C**) Small RNA bioanalyzer chip result of RNA extracted from a two-step FLAG-RNAPII IP either from HEK293 cell (negative control) or from FLAG-SCAF4 cells. (**D**) Amount of RNA purified from RNAPII IP (single IP), SCAF4-RNAPII (double IP) and SCAF8-RNAPII (double IP), respectively. RNA concentrations were

measured by Qubit HS RNA assay. Data is shown for 3 biological replicates. For each sample a starting material of 15 x 15 cm dishes were used.

Supplemental Fig. 4. RNAPII ELCAP-seq profiles compared to ChIP-seq data.

Comparison of RNAPII ChIP-seq[2] with RNAPII ELCAP-seq (single IP). Both IPs are performed with the same antibody (4H8 monoclonal) against the phosphorylated RNAPII CTD. Data is shown both at the read level (top) and single nucleotide resolution (bottom). Zoom-in below highlights the RNAPII ChIP-seq and RNAPII ELCAP-seq (single IP) data for the ALDH7A1 gene.

Supplemental Fig. 5. Transcriptionally engaged RNAPII, SCAF4-RNAPII and SCAF8-RNAPII ELCAP-seq profiles.

(**A**) ELCAP-seq unnormalized genome browser track of single nucleotide resolution data for transcriptionally engaged RNAPII (single IP), SCAF4-RNAPII (double IP), SCAF8-RNAPII (double IP). (**B**) Density scaled metagene profiles (top) and heatmaps of transcriptionally engaged RNAPII, SCAF4-RNAPII and SCAF8-RNAPII across protein coding genes (n = 19,919). (**C**) ELCAP-seq coverage of transcriptionally engaged RNAPII (single IP), SCAF4-RNAPII (double IP) and SCAF8-RNAPII (double IP) centred around the transcript end site (TES) for all protein coding genes (n = 19,919). (**D**) ELCAP-seq for RNAPII (single IP), SCAF4-RNAPII (double IP) and SCAF8-RNAPII (double IP) mapping to the UPF1 gene.

Supplemental References

1. Nojima, T., Rebelo, K., Gomes, T., Grosso, A.R., Proudfoot, N.J., and Carmo-Fonseca, M. (2018). RNA Polymerase II Phosphorylated on CTD Serine 5 Interacts with the Spliceosome during Co-transcriptional Splicing. Mol Cell *72*, 369-379 e364. 10.1016/j.molcel.2018.09.004.
2. Zatreanu, D., Han, Z., Mitter, R., Tumini, E., Williams, H., Gregersen, L., Dirac-Svejstrup, A.B., Roma, S., Stewart, A., Aguilera, A., and Svejstrup, J.Q. (2019). Elongation Factor TFIIS Prevents Transcription Stress and R-Loop Accumulation to Maintain Genome Stability. Mol Cell *76*, 57-69 e59. 10.1016/j.molcel.2019.07.037.

Methods S1, related to STAR Methods

ELCAP for sequencing and mass-spectrometry: Step-by-step protocol

Cellular fractionation

1. Grow 2-5 x15cm dishes per condition. If using Flp-In T-REx HEK293 stably expressing Dox-inducible FLAG-tagged proteins induce expression overnight by the addition of 1 µg/mL doxycycline.
2. Harvested cells by scraping in 2 mL ice-cold PBS per 15 cm dish. Pellet cells by centrifugation at 1,500 rpm for 5 min at 4 ºC.
3. Wash pellet once in ice-cold PBS. Either snap freeze cell pellet in liquid nitrogen and store at -80 ºC or proceed to cellular fractionation.
4. Resuspend cell pellet in 2 pellet volumes of hypotonic buffer (10 mM HEPES pH 7.5, 10 mM KCl, 1.5 mM $MgCl_2$, 20 mM NEM (E3876, N-ethylmaleimide, Merck), incubated on ice for 15 min and dounce homogenize using 20 strokes with a loose pestle at end of incubation.
5. Pellet nuclei by centrifugation at 3,900 rpm for 15 min at 4 ºC and collected supernatant as cytoplasmic fraction.
6. Resuspend the remaining pellet in 2 pellet volumes (original cell pellet volume) nucleoplasmic extraction buffer (20 mM HEPES pH 7.9, 1.5 mM $MgCl_2$, 150 mM potassium acetate, 10 % (v/v) glycerol and 0.05 % (v/v) NP-40), incubated on ice for 20 min.
7. Centrifuge at 20,000g for 20 min at 4 ºC and remove supernatant as nucleoplasmic fraction. *Optional: This can be pooled with cytoplasmic fraction to obtain a combined soluble fraction.*
8. Resuspend the remaining pellet in chromatin digestion buffer (20 mM HEPES pH 7.9, 1.5 mM $MgCl_2$, 10 % (v/v) glycerol, 150 mM NaCl, 0.1 % (v/v) NP-40 and 250 U/mL Benzonase (Merck Millipore, 70746-4)) and incubate rotating for 1 hour at 4 ºC.

9. Centrifuge Benzonase digested samples at 20,000g for 20 min at 4 ºC and collect supernatant as chromatin fraction.

Immunoprecipitations

1. For each sample (double IP) consisting of approximately 3 mL of chromatin extract, wash 100 uL anti-FLAG M2 Affinity Gel (A2220, Merck) three times in ice-cold PBS containing 0.05% NP-40. After the final wash resuspend in twice the original bead volume.
2. If chromatin extracts have been frozen prior to IP, re-clear extracts by 20 min centrifugation at 20,000g at 4 ºC and collect supernatant as chromatin fraction for IPs.
3. Add 200 uL bead slurry (washed FLAG beads) to 3 mL of chromatin extracts and incubate at 4 ºC for 1.5 h on a rotating wheel.
4. Wash bead 4 times with 5 mL IP wash buffer (150 mM NaCl, 20 mM Tris-HCl pH 7.5, 1.5 mM $MgCl_2$, 3 mM EDTA, 10 % (v/v) glycerol, 0.1 % (v/v) NP-40, phosphatase inhibitors (PhosSTOP, 04906837001, Merck) and protease inhibitor cocktail, 05056489001, Merck)). Transfer beads to a 15 mL falcon tube if not already in a falcon tube, centrifuge at 700 g for 5 min, remove supernatant without touching the pelleted beads. After the final wash resuspend in 200-500uL and transfer to a spin column (Thermo Fisher Scientific, 69705) placed in a 2 mL tube.
5. Wash twice on a spin column (Thermo Fisher Scientific, 69705). Add 200 uL wash buffer per wash, centrifuge at 700 g for 2 min at 4 ºC and discard flow-through.
6. After the final wash, add a stopper (supplied with spin columns) to the bottom of the spin column followed by addition of 300 uL 1 mg/mL 3xFLAG peptide dissolved in IP wash buffer.
7. Incubate beads with FLAG-peptide elution buffer for 1 hour at 4 ºC on a rotating wheel.
8. To confirm immunoprecipitation of FLAG-tagged proteins for the first IP step, save 5% of the FLAG elutions for later to run on an SDS-PAGE for western blot.

9. Dilute the remaining FLAG elution to 1mL per sample by addition of IP wash buffer and use for the subsequent RNAPII immunoprecipitation.
10. For each sample wash 50 uL Dynabeads Protein G (10004D, Thermo) 3 times in PBS, 0.05% NP-40 and incubated with 5 ug of RNAPII 4H8 antibody in a total volume of 1.2 mL for 2 hrs at room temperature.
11. Wash 4H8-conjugated beads 3 times in PBS, 0.05% NP-40, resuspended in 100 uL PBS, 0.05% NP-40 and added to the FLAG elution for the first IP step.
12. Incubate samples 2 hrs at 4 ^{o}C on a rotating wheel.
13. Wash beads on a magnetic stand 5 times in IP wash buffer.
14. To confirm double IP of RNAPII, save 5% of the beads removed for a western blot control of the immunoprecipitation.
15. Proceed with remaining bead pellet for ELCAP-seq or ELCAP-MS as described below.

Preparation of RNA for sequencing (ELCAP-seq)

1. Use the remaining beads from the double IP directly for RNA extraction by addition of 300 uL RNA extraction mix: consisting of 100 uL IP wash buffer + 100 uL Zymo RNA lysis buffer (R1050, Zymo Research Quick-RNA MicroPrep) + 100 uL 100% ethanol) directly to the dry beads.
2. Incubated beads 2 min with the RNA extraction mix at room temperature and place back on the magnetic stand.
3. Transfer supernatant containing the RNA to a new tube and use for isolation of both small (17-200nt RNA) and >200nt RNA using the Zymo Research Quick-RNA MicroPrep (R1050) accordingly to the manufacturer's instructions. Elute purified RNA in 15 uL RNase-free water.
4. To assess size distribution of purified RNA, run 1-3 uL on a small RNA bioanalyzer chip (2100 Bioanalyzer Agilent).
5. Measure RNA concentration by Qubit/RNA HS assay (Q32852, Thermo) accordingly to the manufacturer's instructions.

6. Use 5 uL of isolated RNA for sequencing library prep using the NEBNext Multiplex Small RNA Library Prep kit for Illumina (E7300, NEB). There is no need for end-repair of the RNA prior to the library prep as the Benzonase generated ends are compatible with adapter ligations.
7. Amplify libraries using 9-12 PCR cycles (accordingly to input material, consult the NEBNext Multiplex Small RNA Library Prep manual).
8. Run PCR products a 6 % Novex TBE gel (EC6265BOX, Thermo). Stain gel with SYBR Gold (S11494, Thermo) for 10 min and cut out product within the size range of 140-230 bp (corresponding to an insert size range of 20-90 nt).
9. Crush Gel slices with a RNase-free single-use pestle (12-141-364, Fisher Scientific) and incubated in 250 uL gel elution buffer (supplied with NEBNext kit) for 2 hrs at room temperature.
10. Transfer gel pieces to a Spin-X gel filtration column (CLS8160, Merck) and centrifuged for 2 min at 13.000 rpm.
11. Collect flow-through and precipitate DNA by addition of 750 uL 100 % ethanol, 25 uL 3 M sodium acetate pH 5.5 and 1 uL linear acrylamide (supplied with NEBNext kit).
12. Incubate overnight at -20 ºC.
13. The following day, centrifuge samples at 13.000 rpm for 30 min at 4 ºC.
14. Wash pellets in 80% ethanol (make fresh), air-dry and resuspend in 10 uL TE buffer.
15. Measure DNA concentration of the PCR library by Qubit/HS dsDNA kit (Q32851, Thermo).
16. Preform library QC to confirm size range on an Agilent 4200 TapeStation.
17. Sequence samples on a HiSeq4000 (Illumina) (SE75 run) or similar.

Proteomics of RNAPII complexes (ELCAP-MS)

1. Use the remaining beads from the single or double IP step above to eluate bound proteins: Add 50 uL glycine elution buffer (100 mM glycine pH 2.4) directly to dry beads, incubate 5 min at room temperature and vortex.

2. Transfer supernatant containing eluted proteins to a new tube and neutralise by addition of 25 uL 1 M Tris pH 8.8.
3. Add an equal volume of 2x SDS containing loading buffer run samples on SDS-PAGE.
4. Run gel at 100 V for approximately 10 min to allow samples to migrate 2 cm into the gel. Cut out entire gel piece containing stained proteins.
5. Preform in-gel digested with trypsin, using a Janus Automated Workstation (Perkin Elmer).
6. Analyze peptides using an LTQ Orbitrap-Velos mass spectrometer coupled to an Ultimate3000 HPLC equipped with an EASY-Spray nanosource (Thermo Fisher Scientific) or similar setup.

ELCAP-seq analysis

1. Trim reds using TrimGalore v0.4.4 (Martin, 2011). Discard reads < 10 bp in length and those with a maximum error rate > 0.05.
2. Align remaining reads using HISAT2 v2.0.4 against the GRCh38 genome build in a strand-specific manner, allowing for at most 5 distinct primary alignments for each read (Kim et al., 2015).
3. Sort and index reads using SAMtools (Handsaker et al., 2009). Remove multi-mapping reads.
4. Remove duplicate reads using Picard.
5. Create bedgraph files directly from the filtered single-end BAM files using BEDTools (Quinlan et al., 2010) and thereafter bigwig files using bedGraphToBigWig (Kent et al., 2010). Create bigwig files at maximum (i.e., single-bp, not binned) resolution.
6. For feature profiles (metagene plots, TSS and pA centred plots), density scale data after bin size selection to allow comparison of binding behaviour between RNAPII reference (single IP) and the RNAPII subpopulation (double IP).

Troubleshooting tips

Issue	Solution
No FLAG-IP	Check expression of FLAG-tagged protein in input samples using WB. For dox-inducible cell lines make sure dox induction is carried out for a least 14 hrs.<br><br>If FLAG protein is expressed in input samples but fails to IP, try tagging the protein of interest at other terminus or adding a linker between the tag and protein.
No RNAPII IP	Make sure 4H8 is conjugated to beads.<br><br>Run a silver stain with 1/100 dilution input sample, 1/100 dilution of unbound and a few uL of the IP sample.
Both RNAPII single IP and FLAG single IP works, but not double FLAg-RNAPII IP	Check that your factor of interest is associated with elongating RNAPII e.i. IPed by the anti-4H8 antibody. See Table S1.<br><br>If your factor of interest interacts exclusively with unphosphorylated RNAPII, consider using the 8WG16 antibody for the single RNAPII refence IP instead.
No RNA co-IPed	Take care to work RNase-free during the RNA extraction as small RNA fragments are no longer protected from degradation by the RNAPII complex.<br><br>Make sure that the double FLAG-RNAPII IP works and use the single RNAPII 4H8 IP as a positive control compared to a double FLAG-RNAPII-4H8 IP from cells not expressing a FLAG-tagged protein as a negative control.
RNA from single RNAPII IP samples, but not in factor-specific IP	Make sure that the single FLAG IP is efficient.<br><br>If background is an issue, shorten the incubation with the FLAG beads and scale down the amount of FLAG beads/extract volume.<br><br>If the control is clean, but the FLAG-IP yield for the factor of interest is low consider scaling up the amount of input material. Make sure expression of FLAG-tagged protein (see point above) is alright.
RNA sizes > 100 nt in IP	Make sure Benzonase enzyme has been stored correctly. Keep aliquots and avoid extended time outside freezer.<br><br>Increase incubation time with Benzonase to ensure complete digestion of unprotected DNA/RNA.
Appearance of adapter-adapter ligations peak after gel purification	Use a lower dilution of the RNA adapter (1:5 or 1:10) for low RNA input samples during the library prep.

	If issues persist, consider introducing a AMPure bead purification prior to the TBE gel purification.
The DNA library sizes appears as a smear on the TBE gel	The appearance of large fragment sizes can be due to overamplification of the library. Decrease the number of PCR cycles and if possible, test the number of optimal cycles from a test IP sample removing samples at cycle 6, 8, 10, and 12.
