# Supplementary material for: Elongation factor-specific capture of RNA polymerase II complexes
Source: Cell Rep Methods. 2022 Dec 9;2(12):100368. doi: 10.1016/j.crmeth.2022.100368 (PMC9795356; doi:10.1016/j.crmeth.2022.100368)
Supplement: Document S2. Article plus supplemental information [file mmc4.pdf]

Cell Reports Methods

Article

Elongation factor-specific capture of RNA polymerase II complexes

Graphical abstract

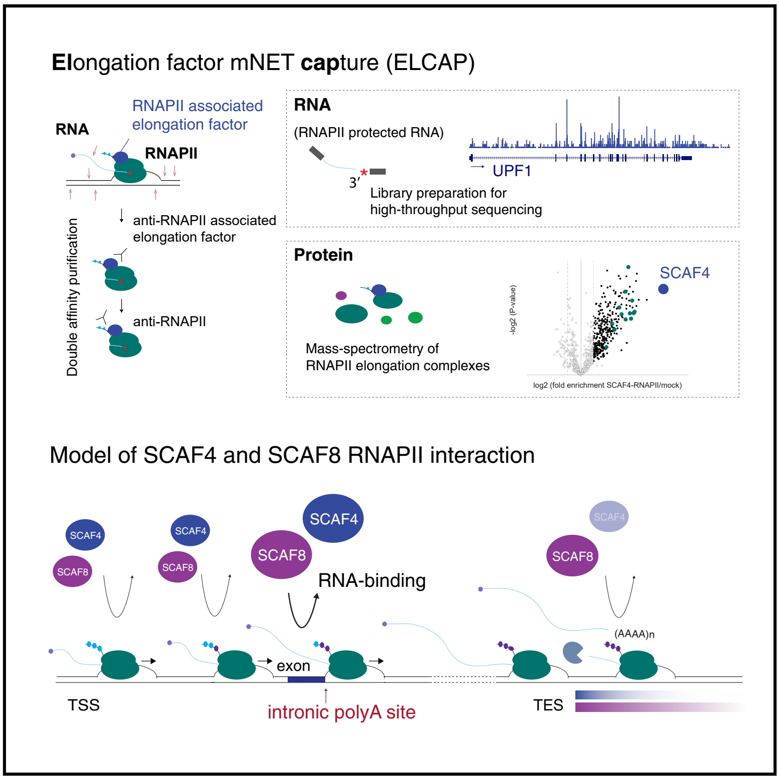

Authors

Lea H. Gregersen, Richard Mitter, Jesper Q. Svejstrup

Correspondence

leag@sund.ku.dk (L.H.G.),
jsvejstrup@sund.ku.dk (J.Q.S.)

In brief

Gregersen et al. develop ELCAP, an affinity purification method that enables capture of factor-specific RNA polymerase II elongation complexes for single-nucleotide occupancy profiling and proteomics.

Highlights

- ELCAP captures RNAPII subcomplexes to facilitate studies of RNAPII-bound factors
- Provides single-nucleotide binding profiles and proteomics characterization
- Highlights overlapping and separate features of RNAPII-bound SCAF4 and SCAF8

Gregersen et al., 2022, Cell Reports Methods 2, 100368
December 19, 2022 © 2022 The Authors.
https://doi.org/10.1016/j.crmeth.2022.100368

CellPress

Cell Reports Methods

CellPress
OPEN ACCESS

Article

Elongation factor-specific capture of RNA polymerase II complexes

Lea H. Gregersen,[1,*] Richard Mitter,[2] and Jesper Q. Svejstrup[1,3,4,*]
[1]Department of Cellular and Molecular Medicine, University of Copenhagen, Blegdamsvej 3B, 2200 Copenhagen, Denmark
[2]Bioinformatics and Biostatistics, The Francis Crick Institute, 1 Midland Road, London NW1 1AT, UK
[3]Mechanisms of Transcription Laboratory, The Francis Crick Institute, 1 Midland Road, London NW1 1AT, UK
[4]Lead contact
*Correspondence: leag@sund.ku.dk (L.H.G.), jsvejstrup@sund.ku.dk (J.Q.S.)
https://doi.org/10.1016/j.crmeth.2022.100368

MOTIVATION Recently developed approaches allow profiling of RNAPII occupancy at single-nucleotide resolution. However, these methodologies are currently not directly applicable to profile RNAPII subcomplexes co-bound by specific regulatory factors. Here, we have optimized chromatin extraction procedures to preserve interactions between RNAPII and its co-bound factors and used them for a double-affinity purification protocol to capture RNAPII subcomplexes for RNA-seq and proteomics. As we capture hundreds of RNAPII-associated factors in our reference RNAPII proteomics data, we reason that this method can be widely applicable to study RNAPII-associated factors.

SUMMARY

Transcription of protein-coding genes is regulated by dynamic association of co-factors with RNA polymerase II (RNAPII). The function of these factors and their relationship with RNAPII is often poorly understood. Here, we present an approach for elongation-factor-specific mNET capture (ELCAP) of RNAPII complexes for sequencing and mass spectrometry analysis aimed at investigating the function of such RNAPII regulatory proteins. As proof of principle, we apply ELCAP to the RNAPII-associated proteins SCAF4 and SCAF8, which share an essential role as mRNA anti-terminators but have individual roles at the 3′ end of genes. Mass spectrometry analysis shows that both SCAF4 and SCAF8 are part of RNAPII elongation complexes containing 3′ end processing factors but depleted of splicing components. Importantly, the ELCAP sequencing (ELCAP-seq) profiles of SCAF4- and SCAF8-RNAPII complexes nicely reflect their function as mRNA-anti-terminators and their competing functions at the end of genes, where they prevent or promote transcriptional readthrough.

INTRODUCTION

Transcription of protein-coding genes by RNA polymerase II (-RNAPII) is a dynamic and highly regulated process. Much of the regulation is dependent on the C-terminal domain (CTD) of the largest RNAPII subunit RPB1; in humans, the CTD consists of 52 heptad repeats with the consensus sequence Tyr_1-Ser_2-Pro_3-Thr_4-Ser_5-Pro_6-Ser_7.[3,4] The CTD is dynamically phosphorylated during the transcription cycle and serves as a binding platform for RNAPII-associated factors, often with specific preferences regarding the CTD phosphorylation signature. These specificities are crucial for correct regulation of transcript elongation and co-transcriptional processing of the pre-mRNA transcript.[3–7] Previously, the occupancy of RNAPII across genes was often determined by chromatin immunoprecipitation combined with next-generation sequencing (ChIP-seq).[8–11] More recently, transient transcriptome sequencing (TT-seq) has been used to map the position of RNAPII transcription activity,[1,12,13] while native elongating transcript sequencing (NET-seq) of chromatin-associated RNAs[14] or mammalian NET-seq (mNET-seq) has been employed to obtain nucleotide-resolution information on the position of RNAPII in its different phosphorylated forms.[15–17] Importantly, however, RNAPII CTD phosphorylation does not itself regulate transcription or co-transcriptional RNA processing but instead enables the recruitment of a number of different, specific co-factors that regulate RNAPII transcriptional initiation, pause-release, transcript elongation, co-transcriptional RNA processing, and termination.[3,10,18,19]

We have previously shown that the CTD-associated, RNA-binding proteins SCAF4 and SCAF8 share an essential, redundant function as mRNA transcript anti-terminators in human cells.[1] In addition, they have individual roles. SCAF8 thus functions as a general RNAPII elongation factor, while SCAF4 promotes transcription termination downstream of the transcript end site (TES).

This is an open access article under the CC BY license (http://creativecommons.org/licenses/by/4.0/).

Despite these differences, the RNA-binding profiles for SCAF4 and SCAF8 identified by photoactivatable ribonucleoside-enhanced crosslinking and immunoprecipitation (PAR-CLIP) experiments were surprisingly similar.[1] We were therefore interested in instead determining whether the interaction of SCAF4 and SCAF8 with distinct RNAPII elongation subcomplexes might explain their different roles in regulation of transcription elongation and termination. For this purpose, we established a generally applicable approach to capture elongating RNAPII complexes but, specifically, those subcomplexes that are bound by a co-factor—in this case, an SCAF protein. For this purpose, we used two-step IP starting from nuclease-treated chromatin extracts to enable the capture of RNAPII complexes. This approach, termed elongation-factor-specific mNET capture (ELCAP), was then used with sequencing and mass spectrometry to analyze the content and behavior of SCAF4-bound RNAPII and SCAF8-bound RNAPII complexes. In accordance with their shared role as mRNA anti-terminators, SCAF4- and SCAF8-bound RNAPII complexes display similar binding profiles within the gene body and interact with RNAPII complexes bound by elongation factors. However, at the same time, we find markedly different binding profile around the TES in SCAF-regulated genes, supporting their different role during transcriptional termination.

RESULTS

We reasoned that double-affinity purification of RNAPII complexes with an associated factor would allow an investigation of the significance of the interaction. As we wanted to map the position of such complexes in genes with high precision across the genome, we used the mNET-seq protocol as a starting point. mNET-seq is based on isolation of chromatin-bound RNAPII elongation complexes using stringent conditions,[17] so we first tested if these conditions allow the isolation of RNAPII complexes associated with SCAF4 and SCAF8. Unfortunately, most SCAF4 and SCAF8 protein was released from chromatin with a large fraction of phosphorylated RNAPII during this extraction procedure (Figures 1A and 1B). We therefore modified the protocol (see STAR Methods and Methods S1 for a detailed step-by-step protocol). Like in the mNET-seq protocol, we initially carried out stepwise, cellular fractionation: cytoplasmic proteins were removed from intact nuclei by hypotonic lysis, followed by extraction of nucleoplasmic proteins using 0.05% NP-40. The remaining chromatin pellet was then dissolved in 150 mM NaCl and 0.1% NP-40, and the chromatin-bound proteins were released by DNA/RNA digestion. For our approach, we replaced micrococcal nuclease (MNase) with Benzonase, which we have previously used to purify RNAPII complexes from chromatin as it results in excellent recovery of transcriptionally engaged RNAPII.[1,20,21] Importantly, like MNase digestion, Benzonase treatment allows the recovery of the short, nascent RNA fragments protected by the RNAPII elongation complexes, which are suitable for deep sequencing (Figures 1C and S1A). An additional advantage of Benzonase digestion is that, in contrast to MNase digestion, it can take place directly in the chromatin extraction buffer, alleviating the need for a separate nuclease treatment step in a new buffer. Together, these changes resulted in excellent recovery of soluble, phosphorylated RNAPII and SCAF4- and SCAF8-bound RNAPII complexes (Figure 2A). Importantly, compared with mNET-seq, the amount of RNAPII in the starting material for the IP step was markedly increased using this modified extraction protocol without detectable background in our control IP (Figures 1B and 2A).

To investigate how well the ELCAP protocol captured specific RNAPII-associated proteins, we performed mass spectrometry analysis of the proteins co-purified with RNAPII from our single IP (RNAPII ELCAP-MS). For this purpose, a label-free approach of two biological replicates, each injected in triplicate, was used. As a negative control for the RNAPII IP, we performed an immunoglobulin G (IgG; mock) IP (Figure S1B and S1C). The data obtained showed a high degree of reproducibility of enriched proteins associated with RNAPII from the biological replicates (Figure S1C). We detected hundreds of proteins associated with the transcriptionally engaged RNAPII with a log2 fold change >2 and a −log2 p value >2 (Figure 2B; Table S1). As expected, the interactome is highly enriched for known RNAPII-binding proteins with functions in elongation, splicing, and termination, validating the approach. We also compared our data with previous MS of mNET IPs of RNAPII Ser2P and Ser5P (mNET-MS).[15] Gratifyingly, we observed a greater enrichment of RNAPII-associated factors with our RNAPII ELCAP-MS (single IP) protocol (Figures S2A–S2D). While proteins that were identified only by RNAPII ELCAP-MS and not by mNET-MS are enriched in Gene Ontology terms related to transcription, chromatin remodeling, and RNA processing, most proteins identified by mNET-MS, but that were not found by RNAPII ELCAP-MS, are histone or ribosomal proteins (Figure S2C). Notably, while SCAF4 and SCAF8 were not detected at all or only just detected in mNET-MS with low log2 fold changes compared with control, we found them both reproducibly enriched in our RNAPII ELCAP-MS (Figures S1C, S2A, and S2B). This indicates that the RNAPII (single IP) ELCAP-MS protocol is well suited to capture co-factors of transcriptionally engaged RNAPII complexes.

The single-affinity purifications showed that while SCAF4 IPs are highly enriched in phosphorylated RNAPII, SCAF4 is not enriched to the same extent in an RNAPII IP (Figure 1B and S3A). This suggests that while most SCAF4 protein is bound to RNAPII, only a small fraction of the total pool of transcriptionally engaged RNAPII is bound to SCAF4. For efficient double-affinity purification, SCAF4 or SCAF8 was therefore affinity enriched first, followed by RNAPII IP (Figures 1A, 2A, and S3B). Both SCAF4 and SCAF8 were reproducibly and significantly enriched by the double IP purification protocol, first using FLAG-SCAF4 or FLAG-SCAF8 affinity purification, followed by affinity purification of transcriptionally engaged RNAPII using the 4H8 antibody recognizing the RNAPII-phosphorylated CTD (Figure 2A). In the first purification step, most of the chromatin-associated SCAF4 or SCAF8 complexes are depleted from the flow through (Figure 2A), while in the second purification step targeting CTD-phosphorylated RNAPII, a significant proportion of SCAF4 or SCAF8 isolated in the first step is recovered (Figures 2A and S3B).

To investigate whether the two-step affinity-purification (double IP) approach captures SCAF4- and SCAF8-bound RNAPII complexes and their associated factors, we again performed MS analysis (Figures 2C and 2D). As a negative control for the double-affinity purification of SCAF4 or SCAF8 bound to RNAPII, we performed an initial FLAG IP from control cell lines

CellPress
OPEN ACCESS

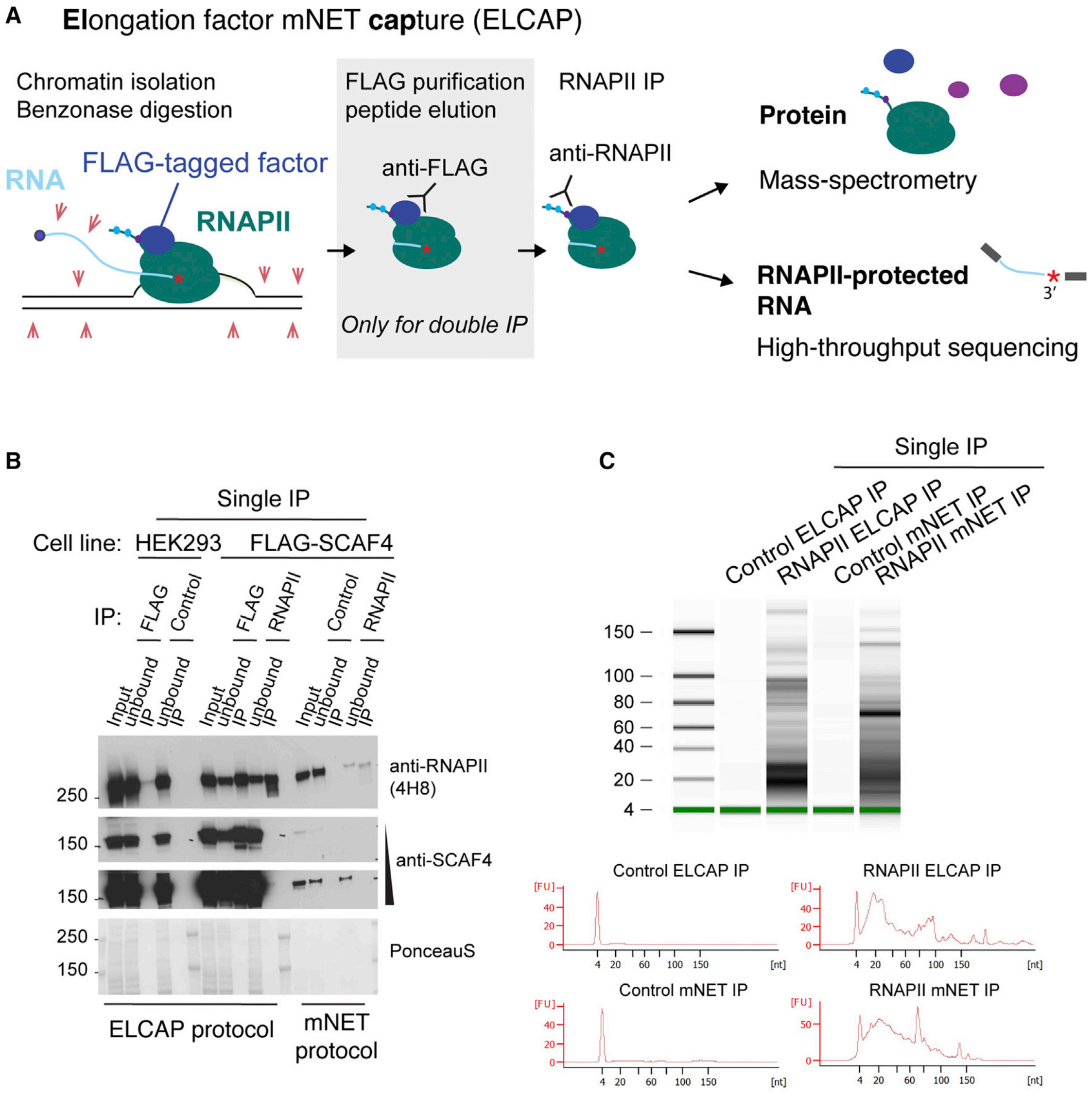

Figure 1. Development of ELCAP

(A) Outline of the ELCAP protocol for obtaining factor-bound RNAPII complexes for proteomics or sequencing. Isolated chromatin is Benzonase treated to digest (red arrows) any unprotected DNA and RNA. RNAPII is either directly immunoprecipitated (single IP) using an antibody against the phosphorylated RNAPII CTD or the RNAPII-bound factors SCAF4 or SCAF8 are purified using an initial FLAG affinity purification followed by a second purification step against RNAPII (double IP). The RNAPII elongation complex proteins are processed for label-free mass spectrometry to determine the protein composition of elongation complexes. In parallel, the position of RNAPII is determined at nucleotide resolution from the 3′ end of the protected RNA fragment.

(B) Single-affinity purification of RNAPII and single-step FLAG-SCAF4 IP using the ELCAP chromatin extraction procedure or a single-affinity purification of RNAPII using a mNET-seq chromatin extraction procedure as described previously.[22] For each sample, the input material was harvested from the same amount of starting material (5 × 15 cm dishes) to allow a direct comparison.

(C) Small RNA bioanalyzer chip result of RNA extracted from a single-step RNAPII IP from chromatin extracted and nuclease treated according to either the ELCAP or mNET-seq procedure.

not expressing a FLAG-tagged SCAF protein, followed by a subsequent RNAPII IP. Samples were prepared as biological duplicates, and each sample was injected in triplicates. As expected, we identified fewer interactors in the double IP of either SCAF4- or SCAF8-associated RNAPII compared with the single RNAPII IP, but reassuringly, numerous known RNAPII-associated factors were highly enriched in both double IPs (Figures 2C and 2D).

SCAF4 and SCAF8 bind RNAPII complexes containing elongation and 3′ end processing factors

We now analyzed the proteomic data to retrieve information about how SCAF4- and SCAF8-bound RNAPII complexes distinguish themselves from the larger pool of RNAPII complexes. Looking at factors specifically enriched in the SCAF4-RNAPII or SCAF8-RNAPII double IPs compared with

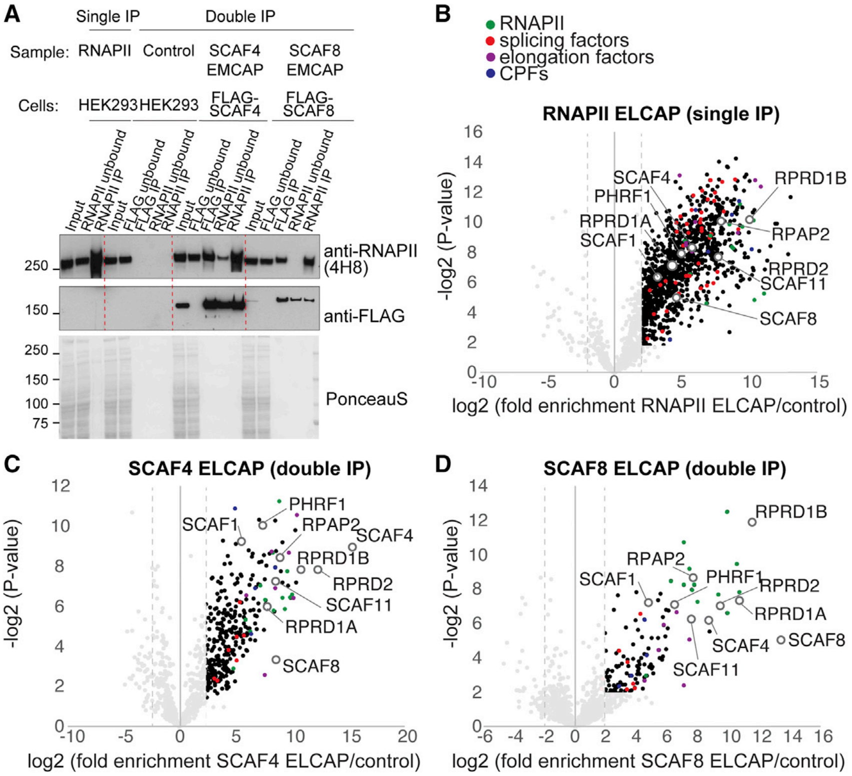

Figure 2. ELCAP-MS of SCAF4- and SCAF8-bound RNAPII complexes

(A) Western blot control of RNAPII IPs (single IP) and FLAG-SCAF4 or -SCAF8 followed by RNAPII IP (double IP). As a negative control, a FLAG IP and a subsequent RNAPII IP from HEK293 cells not expressing any FLAG-epitope-tagged proteins were used.

(B) Volcano plot of proteins enriched in RNAPII ELCAP (single IP) from two biological replicates (triplicate injection).

(C and D) Volcano plot of proteins enriched in SCAF4 ELCAP (C) and SCAF8 ELCAP (D) double IPs from two biological replicates (triplicate injection).

single RNAPII IP interactome, we observed that several RNAPII CTD-associated factors were preferentially enriched after SCAF4- or SCAF8-RNAPII double-affinity IP (Figures 3A and 3B). These include SPT6, IWS1, RPRD1A, RPRD1B, RPAP2, RPRD2, RECQL5, and SPT5, as well as the SR-related and CTD-associated factors SCAF1, SCAF11, and PHRF1 (Figures 3A and 3B; Table S1). We also observed an enrichment of cleavage and polyadenylation specificity factor (CPSFs) involved in 3′ end processing (Table S1). The association of CPSFs was stronger in SCAF4-RNAPII complexes (Figures 3A–3D), which agrees with SCAF4's unique role in preventing transcriptional readthrough.[1] Strikingly, we did not see enrichment of known splicing factors or proteins involved in regulation of splicing that were highly enriched in the single RNAPII IPs, such as SF3 factors, pre-mRNA processing factors (PRPFs), serine/arginine rich splicing factors, or factors involved in alternative splicing regulation such as CHERP or MATR3 (Table S1). We also did not find any enrichment of integrator subunits in the SCAF4- or SCAF8-RNAPII IPs, although all members of the integrator complex were highly enriched in the reference RNAPII IP (Table S1). These results are important as they support the idea that SCAF4 and SCAF8 bind (or establish) specific subpopulations of RNAPII complexes rather than associating with a random fraction of transcriptionally engaged RNAPII. Overall, most proteins that were highly enriched in the SCAF4-RNAPII IP were also enriched in the SCAF8-RNAPII IPs, suggesting that SCAF4 and SCAF8 recognize, or are part of, a similar subset of RNAPII elongation complexes (Figures 3C and 3D).

ELCAP-seq efficiently captures RNAPII with high coverage throughout the gene body

Because the nascent RNA inside the elongating RNAPII complex is protected from the nuclease digestion performed during chromatin fractionation, the double-affinity-purified SCAF4- or SCAF8-RNAPII complexes could also be used for RNA extraction and library production (Figures 1A, 1C, S3C, and S3D). As expected, the amount of isolated RNA is smaller from the subpopulation of RNAPII complexes bound by SCAF4 or SCAF8 compared with a single RNAPII IP (Figure S3D). However, in all cases, we could reproducibly obtain enough material for small RNA library preparation for sequencing (ELCAP-seq).

To reduce sequencing costs and simplify the computational analysis, we used single-end sequencing, which still allows the mapping of reads in a strand-specific manner. We developed a pipeline to handle both single-end and paired-end data as input to allow direct comparison of data from ELCAP-seq with published mNET-seq datasets (see STAR Methods for details). We obtained high-resolution profiles of RNAPII binding at a single gene level (Figure 4A). As in mNET-seq, ELCAP-seq profiles provide nucleotide resolution of RNAPII location based on the position of the 3′ end of the protected RNA fragment. The difference in gene resolution between ELCAP-seq profiles and ChIP-seq can be appreciated by comparison with publicly available RNAPII ChIP-seq data (Figure S4). Metagene profiles confirm that ELCAP-seq preferentially captures RNAPII within the gene body (Figure 4B). Indeed, we obtain a higher gene body coverage with the ELCAP-seq approach than that of published total RNAPII mNET-seq profiles (Figures 4B and 4C), in accordance with the excellent enrichment of transcriptionally engaged RNAPII by the optimized Benzonase-based procedure (Figures 1B and 2A). Together, these data show that the ELCAP-seq protocol captures transcriptionally engaged RNAPII complexes at nucleotide resolution with strand information and high coverage across the gene body.

CellPress
OPEN ACCESS

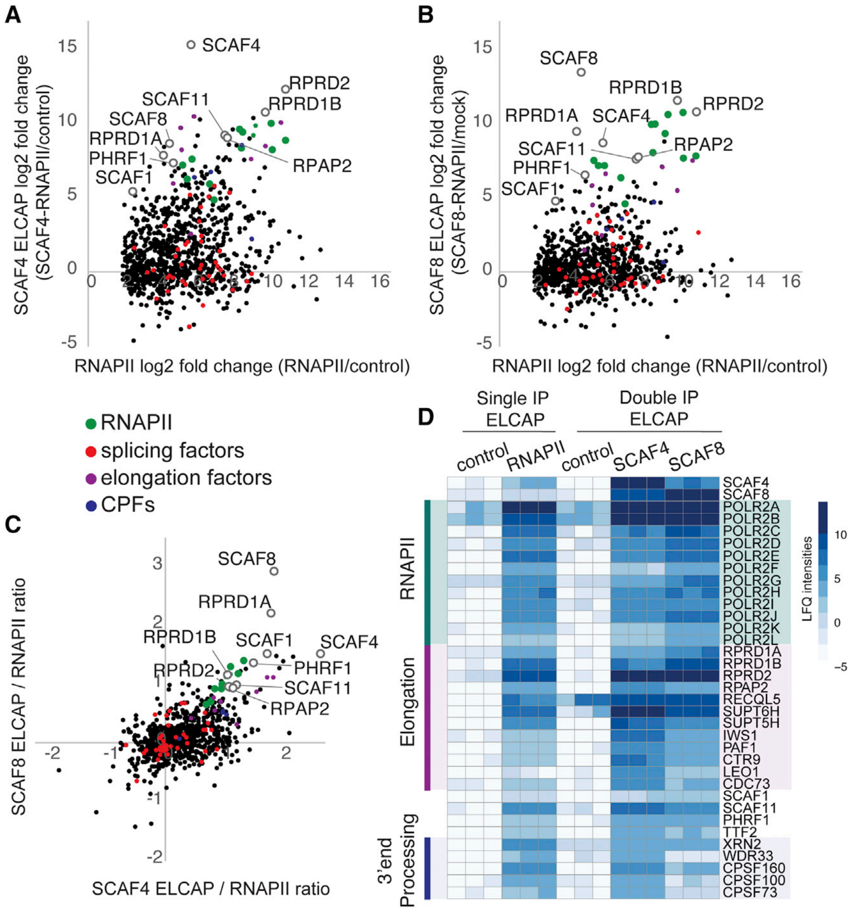

Figure 3. SCAF4- and SCAF8-bound RNAPII complexes are enriched in elongation and 3′ end processing factors

(A) SCAF4 ELCAP for mass spectrometry compared with single IPs of transcriptionally engaged RNAPII. Only proteins with a −log2 p >2 and a log2 fold enrichment >2 in the RNAPII IP were considered. Proteins highlighted are previously identified SCAF4-binding factors.

(B) As in (A) but for SCAF8.

(C) Correlation of proteins identified as part of SCAF4 ELCAP (double IP) and SCAF ELCAP (double IP) subcomplexes.

(D) Heatmap of ELCAP-MS label-free quantitation (LFQ) intensities for selected factors. Data are shown for 3 replicate injections for one of the biological replicates. Single IP ELCAP control is a bead-only IP. Double IP ELCAP control is a FLAG IP from parental HEK293 cells followed by an RNAPII IP.

SCAF4 and SCAF8 bind to transcriptionally engaged RNAPII throughout the gene body and beyond the cleavage and polyadenylation site

We now investigated how the RNAPII profiles change by their association with SCAF4 or SCAF8. Interestingly, the profiles for SCAF4- and SCAF8-associated RNAPII complexes were markedly different from that obtained with RNAPII alone, which represents an average of all RNAPII complexes engaged in transcription. Indeed, RNAPII in SCAF4 or SCAF8 complexes was depleted in the area downstream of the transcription start site (TSS) but highly enriched toward the 3′ end of gene bodies and downstream of the cleavage and polyadenylation (polyA) site (Figure 5A). At first glance, this may seem counterintuitive as we have previously shown that SCAF4 and SCAF8 share an essential function as mRNA anti-terminator proteins that interact with nascent RNA near the 5′ region of the transcripts, where premature termination is suppressed.[1] However, while SCAF4 and SCAF8 perform a critical role in preventing the usage of intronic polyA sites at a subset of genes, they are also important for general regulation of transcription at the 3′ end of genes. Indeed, *SCAF4* single knockouts (KOs) display extended transcriptional readthrough beyond the cleavage and polyA site—an effect that is completely dependent on the presence of SCAF8.[1] We therefore divided our analysis of the ELCAP-seq data into two parts: one focused on the association of RNAPII around early (or cryptic/intronic) polyA sites and another around the canonical cleavage and polyA sites at gene ends to address the two separate functions of SCAFs: the redundant, essential role as mRNA anti-terminators, and their distinct roles in preventing or promoting transcriptional readthrough, respectively.

Binding of SCAF4 and SCAF8 to RNAPII around intronic polyA sites

In considering the significance of RNAPII density peaks, it is important to remember that peaks of RNAPII density within a gene do not signify "high RNAPII activity" in this area but rather pausing or arrest or regions of slow elongation.[23] Our previous work showed that SCAF4 and SCAF8 bind directly to nascent RNA upstream of intronic polyA sites to prevent early mRNA transcript cleavage and subsequent termination,[1] which led us to now ask whether the loading of SCAF proteins onto RNAPII correlates with locally altered pausing or elongation around such sites. To answer this question, we investigated if SCAF4- and SCAF8-RNAPII complexes are generally enriched or depleted around the intronic polyA sites identified as repressed by SCAF4 and SCAF8.[1] Intriguingly, while we previously observed increased binding to nascent RNA of both SCAF4 and SCAF8 upstream of SCAF4- and SCAF8-suppressed intronic polyA sites using PAR-CLIP,[1] a general change in RNAPII location on DNA specifically for SCAF4 or SCAF8 complexes at these intronic polyA sites was not observed (Figure 5B). There was, however, a general tendency to a higher RNAPII density toward the intronic polyA site and a modest decrease after it. This could indicate a slower RNAPII elongation rate or transient RNAPII stalling upstream of the intronic pA site together with a faster RNAPII elongation rate downstream of the intronic polyA site. Of note, the profiles did not change between the SCAF4- and SCAF8-bound complexes

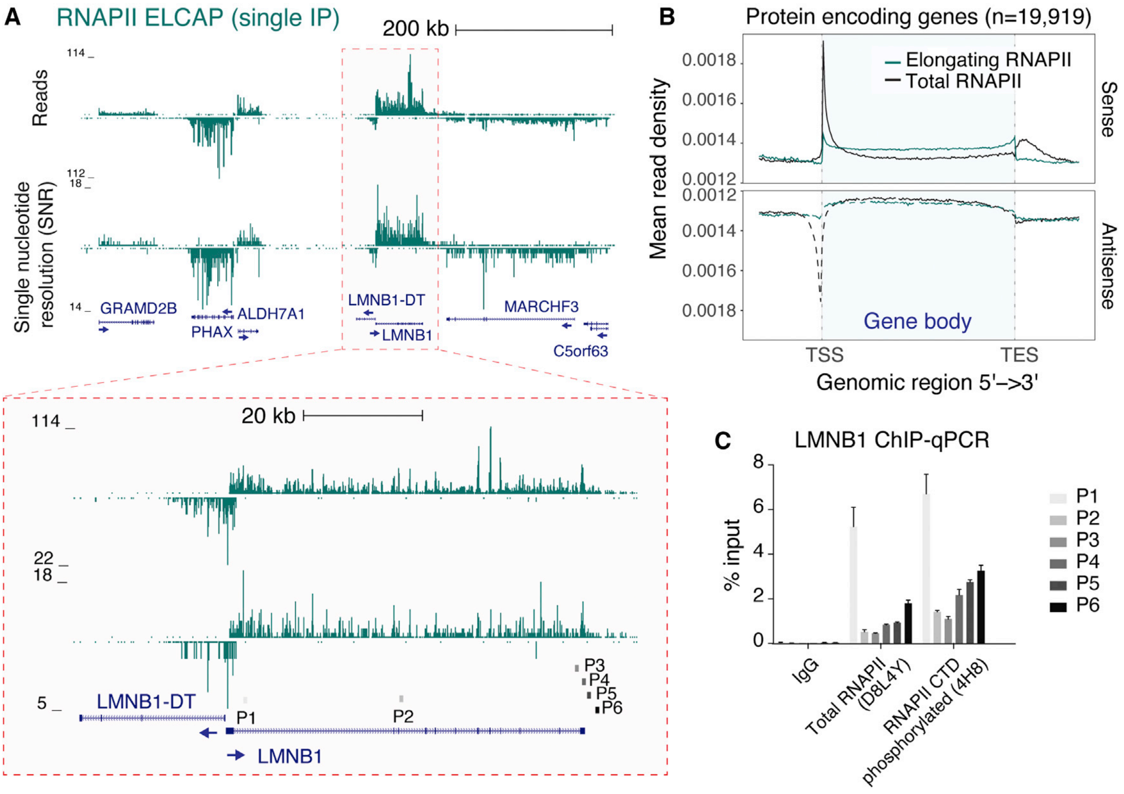

Figure 4. ELCAP-seq provides high-resolution profiles of RNAPII throughout the gene body

(A) Strand-specific transcriptionally engaged RNAPII ELCAP (single IP) unnormalized profiles showing either read-level data or single-nucleotide resolution (SNR) data. Zoom in of the LMNB1 gene shown below with primer positions used for ChIP-qPCR in (C) highlighted.

(B) Metagene profiles of total RNAPII mNET-seq[1] and transcriptionally engaged RNAPII ELCAP-seq for protein-coding genes (n = 19,919). Data have been density scaled.

(C) ChIP-qPCR for LMNB1 (primer positions indicated in A) using either antibodies against total RNAPII (D8L4Y) or elongating CTD phosphorylated RNAPII (4H8). Data are shown as percentage of input for two replicates ± standard derivation (SD). IgG was used as a negative control.

and RNAPII in general, suggesting that SCAF4- and SCAF8-bound RNAPII complexes display the same overall behavior around intronic polyA sites. However, we did notice individual examples, where interesting differences could be observed. These are exemplified by the *DNAJC12* gene, which we previously showed contains a cluster of RNA binding for both SCAF4 and SCAF8 by CLIP, located upstream of intronic polyA sites in exon 3. Use of this early intornic polyA sites is suppressed in wild-type (WT) cells, but in *SCAF4 SCAF8* double-KO cells, increased cleavage at this site gives rise to short mRNA transcript isoforms[1] (Figure 5C). Looking at *DNAJC12*, we observed a strong SCAF4 and SCAF8 ELCAP signal in and immediately after exon 3 just upstream of the intronic polyA site that is repressed by SCAF4 and SCAF8 (Figure 5C). It seems reasonable to speculate that this signal represents a region of increased RNAPII pausing at which SCSF4 and SCAF8 are loaded. As mentioned, we know that both SCAF4 and SCAF8 display increased RNA binding to mRNA transcripts upstream of regulated intronic polyA sites,[1] suggesting that the transient loading of SCAF4 and SCAF8 onto RNAPII serves to get both factors into proximity of nascent RNA transcripts, and when the correct determinant is present in the RNA transcript, such as a sequence motif, the RNA binding of SCAF4 and SCAF8 is stabilized.

Together, the data suggest a general model where the interaction between SCAF4 or SCAF8 with RNAPII around intronic polyA sites is dynamic and transient, although at some sites, like the one in *DNAJC12*, a markedly stronger association of SCAF-RNAPII complexes than for RNAPII can be detected. Interestingly, since we previously detected an enrichment of both SCAF4 and SCAF8 binding to the nascent RNA upstream of intronic affected polyA sites,[1] it is possible that dynamic binding of SCAF4 and SCAF8 to RNAPII allows them to “sample” nascent RNA transcripts as these are being actively transcribed, i.e., that RNAPII “deposits” the SCAF proteins at such sites on RNA to inhibit the activity of transcript cleavage factors (Figure 5D).

Differential binding of SCAF4 and SCAF8 to RNAPII around the 3′ end of genes affected by transcriptional readthrough in SCAF4 KO cells

In agreement with their general role in elongation and termination,[1] the ELCAP-seq profiles suggest that SCAF4 and SCAF8 are associated with RNAPII around and beyond the TES. Indeed,

CellPress
OPEN ACCESS

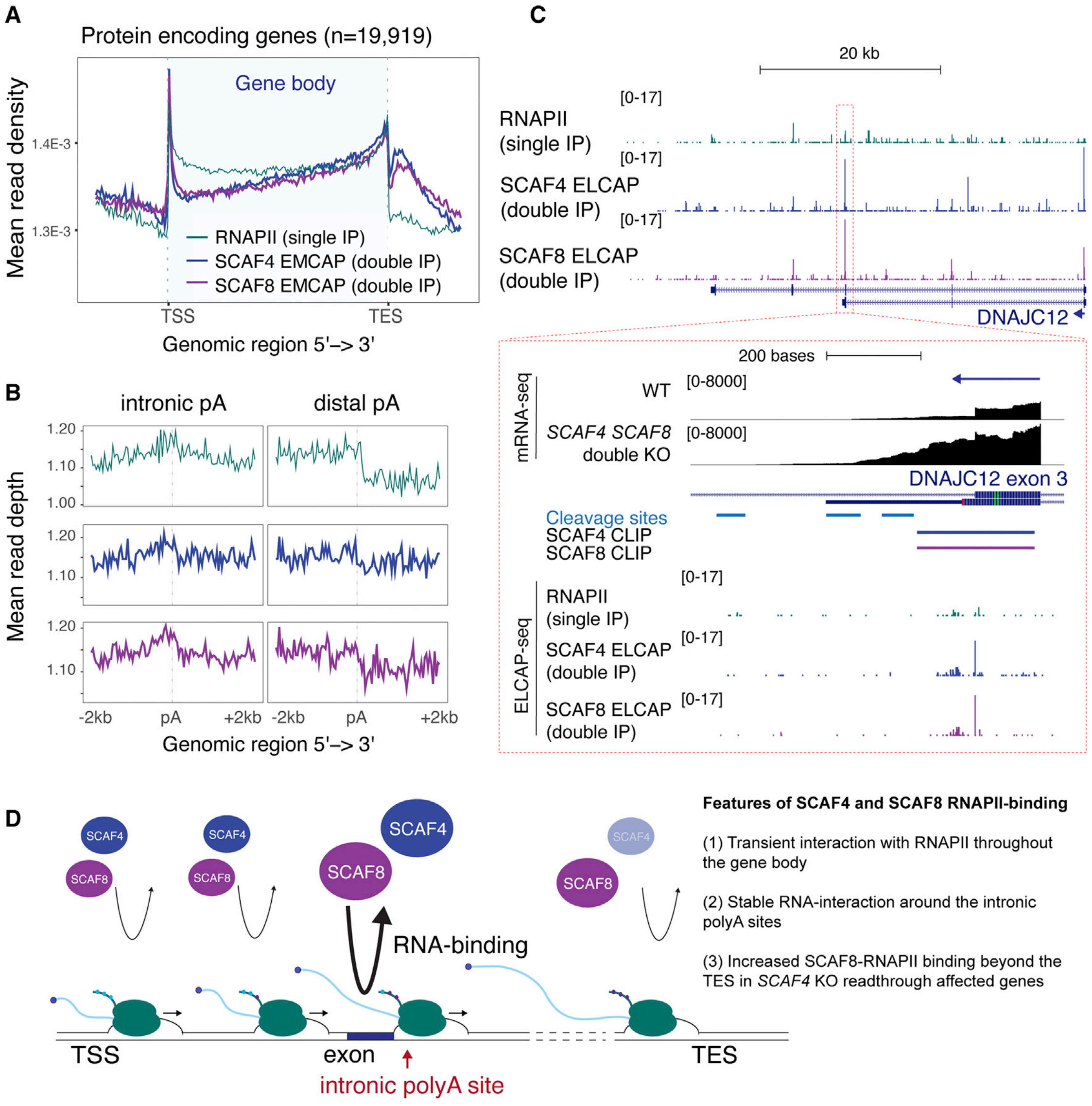

Figure 5. SCAF4 and SCAF8 ELCAP-seq reveals binding to transcriptionally engaged RNAPII throughout the gene body

(A) ELCAP-seq density-scaled metagene profiles of transcriptionally engaged RNAPII, SCAF4-RNAPII, and SCAF8-RNAPII across protein-coding genes (n = 19,919).

(B) As in (A) but showing density ELCAP profiles around intronic polyA sites regulated in a SCAF4- and SCAF8-dependent manner as defined previously[1] and at the distal polyA site for the same set of genes.

(C) ELCAP-seq unnormalized binding profiles of RNAPII (single IP), SCAF4 ELCAP (double IP), and SCAF8 ELCAP (double IP) for the *DNJAC12* gene. In the zoomed-in panel, the ELCAP-seq data are shown together with mRNA-seq data from WT and double *SCAF4 SCAF8* KO cells.[1] Position of the RNA-binding cluster immediately prior to the intronic polyA site identified from CLIP experiments and cleavage site identified from 3′-seq data[1] are indicated.

(D) Model of SCAF4 and SCAF8 binding to RNAPII and nascent RNA. Based on our ELCAP data, both SCAF4 and SCAF8 interact with RNAPII throughout the gene body. While the interaction with RNAPII around SCAF-regulated intronic polyA sites is likely transient, it serves to facilitate a stable interaction between SCAF4/SCAF8 and RNA as identified by CLIP-seq.[1] Beyond the TES of readthrough-affected genes in single *SCAF4* KO, SCAF8 displays a stronger RNAPII interaction than SCAF4. Based on SCAF8's role as a positive elongation factor, we speculate that this could drive the transcriptional readthrough.

while the signal for RNAPII (single IP) itself drops dramatically immediately after the TES, SCAF-bound RNAPII complexes remain abundant for several kb downstream (Figures 5A and S5A–S5D). This indicates that while affinity purification of phosphorylated RNAPII is not capturing RNAPII downstream of the TES as well as within the gene body, the SCAF-bound, phosphorylated RNAPII complexes downstream of the TES are efficiently isolated. An interesting finding from our previous work was that the absence of *SCAF4* alone leads to transcriptional readthrough beyond the TES, sometimes for hundreds of kb.[1] While the dramatic drop in general RNAPII association seen by ELCAP-seq likely signifies rapid termination of a significant

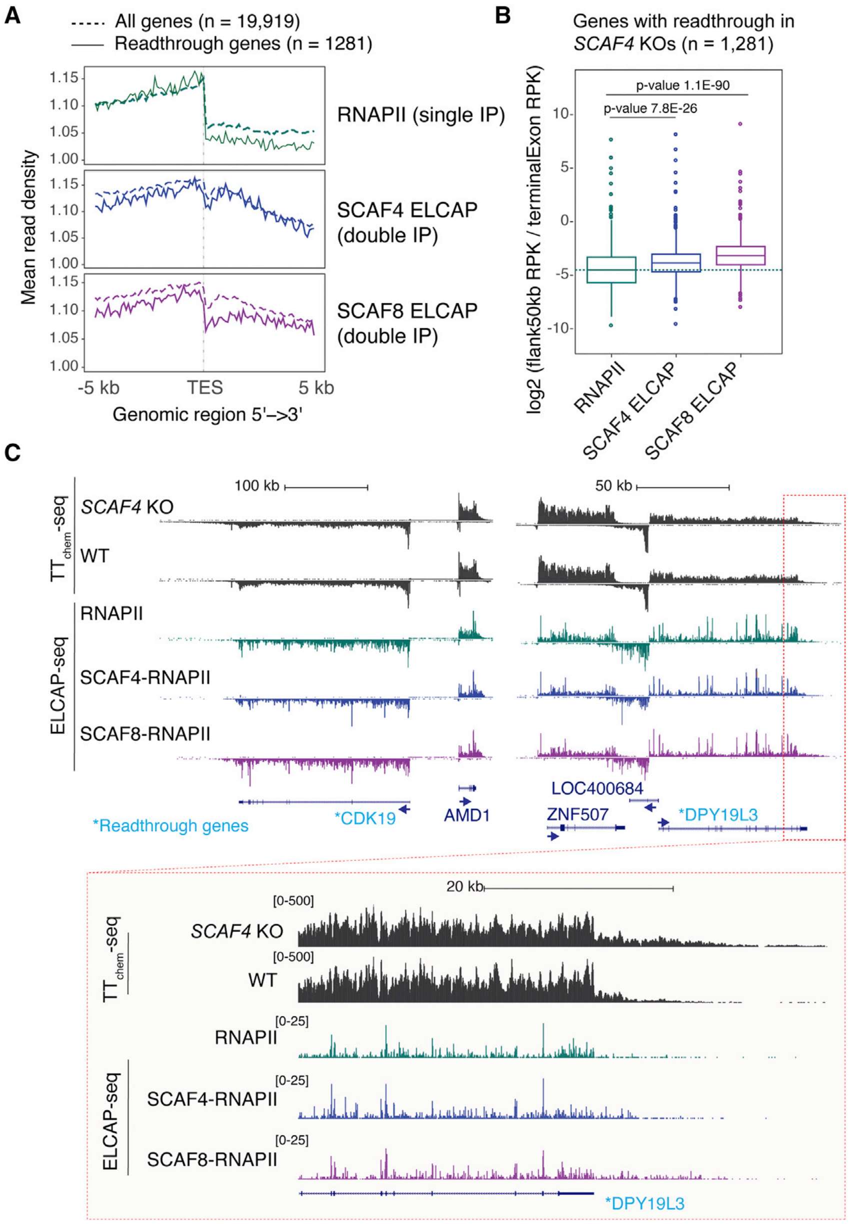

Figure 6. Differential binding of SCAF4 and SCAF8 to RNAPII around the 3′ end of genes affected by SCAF-dependent transcription readthrough

(A) ELCAP-seq density binding profiles around the TES for transcriptionally engaged RNAPII (single IP), SCAF4 ELCAP (double IP), and SCAF8 ELCAP (double IP) for genes with TES readthrough in *SCAF4* KOs (solid line, n = 1281) as defined previously[1] and in all protein-coding genes (dashed line, n = 19,919).

(B) Boxplot of readthrough ratios calculated from ELCAP-seq for genes for genes with nascent transcriptional readthrough in *SCAF4* KOs. Readthrough ratios are calculated from ELCAP-seq occupancy downstream of the most distal transcript end site as the number of reads in the 50 kb regions downstream of the TES relative to the number of reads in the terminal exon. p values were calculated using Wilcoxon ranked t test.

(C) TT_{chem}-seq data from WT and single *SCAF4* KO cells[1] showing nascent transcription beyond the TES of the two readthrough genes CDK19 and DPY19L3 (marked with an asterisk) as well as RNAPII ELCAP-seq (single IP), SCAF4 ELCAP-seq (double IP), and SCAF8 ELCAP-seq for the indication genomic region.

fraction of RNAPII molecules immediately downstream of the TES, the continued association of especially SCAF8 supports of a role for this protein in the termination of a population of RNAPII that escapes the termination signals around the canonical polyA site.

To investigate how SCAF4 and SCAF8 binding to RNAPII correlates with the transcriptional readthrough previously observed in *SCAF4* KO cells,[1] we compared SCAF4- and SCAF8-RNAPII binding profiles around the TES specifically in the genes affected by such readthrough (Figure 6A). Interestingly, while SCAF8 ELCAP-seq profiles in general showed less binding of SCAF8 to RNAPII immediately downstream of the TES (Figure 6A), it showed a higher degree of binding to RNAPII downstream of the TES in readthrough genes (Figures 6B and 6C). We have previously shown that SCAF8 is required for the transcription readthrough observed in *SCAF4* KO cells, as such readthrough is absent in double *SCAF4 SCAF8* KOs.[1] Importantly, in this context, SCAF8 functions as a positive transcription elongation factor to globally promote RNAPII elongation rates,[1] and increased RNAPII elongation rates have been shown to promote transcriptional readthrough in support of a kinetic competition model where fast elongating RNAPII complexes are able to escape XRN2-mediated exonucleolytic RNA decay and RNAPII termination.[24] Together, new and old results thus agree with a model where SCAF8 drives transcription readthrough by promoting RNAPII elongation downstream of the TES (Figure 5D). In contrast, SCAF4 acts to restrict transcription readthrough. Indeed, by ELCAP-seq, SCAF8 and SCAF4 are more highly enriched downstream of the TES on readthrough genes (Figures 6B and 6C).

DISCUSSION

In this study, we used a double-affinity purification approach, ELCAP, for capturing specific elongation complexes to characterize the RNAPII co-factors SCAF4 and SCAF8. By combining ELCAP with MS and next-generation sequencing, we identified the composition of SCAF-bound RNAPII elongation complexes

and mapped the precise position of SCAF-bound RNAPII complexes within the gene body.

As previously shown, profiling of RNAPII elongation complexes using mNET-seq is sufficient to obtain metagene profiles to assess overall RNAPII binding changes and to profile the different RNAPII CTD phosphorylation forms.[15,17] However, we found that the stringent purification conditions used in the mNET-seq protocol were not well suited to capture SCAF-bound RNAPII complexes. We reasoned that if we were to capture subpopulations of RNAPII elongation complexes and obtain high-coverage single gene profiles, the chromatin extraction and nuclease digestion had to be optimized. Using a lower-stringency chromatin extraction procedure, without urea or high detergent levels, we preserved the interaction between RNAPII and its co-factors. This was confirmed by ELCAP proteomics of RNAPII complexes, which obtained specific RNAPII-associated co-factors. This also means that ELCAP can easily be adapted to study these many other RNAPII co-factors.

Single-step IP of elongation factors (TEF-seq) has previously been used to profile Paf1, Spt4, Spt6, and Spt16 across yeast genes.[25,26] This approach did not use a second RNAPII affinity-purification step as used in the ELCAP protocol, which may well be important to fully understand the role of such elongation factors (which are known to, by themselves, also bind DNA, RNA, or nucleosomes). In human cells, the binding of human transcription initiation, elongation, and splicing factors, namely TBP (part of TFIID), SPT6, and SSRP1, was profiled using NET-prism.[27] Like TEF-seq, NET-prism is based on single-step purification against the factor of interest but relies only on DNA digestion of the chromatin extracts; no RNA digestion is carried out prior to the library preparation. For the splicing factor SSRP1, a sequential NET-prism and RNAPII IP was carried out to confirm isolation of RNAPII-bound SSRP1.[27] However, the RNAPII IP was carried out using an antibody recognizing the unphosphorylated CTD of RNAPII (8WG16 antibody), and, consequently, the profile had almost no coverage within the gene body. By contrast, ELCAP-seq achieves high-coverage profiles throughout the gene body both for the general RNAPII ELCAP-seq and for the SCAF4 and SCAF8 ELCAP-seq.

It is worth noting that we previously attempted to perform SCAF4 and SCAF8 ChIP-seq experiments using either a standard formaldehyde or a disuccinimidyl glutarate (DSG) crosslinking approach. However, we were unable to obtain meaningful results with either factor (data not shown). This points to the general usefulness of the ELCAP-seq protocol for studies of factors that do not themselves bind DNA and which interact with RNAPII only during specific transcription events. Indeed, both SCAF4 and SCAF8 ELCAP-seq binding profiles provide excellent high-resolution maps across the gene body and beyond the cleavage and polyA sites, while ELCAP-MS confirmed that both SCAF4 and SCAF8 are found in transcriptionally engaged RNAPII complexes together with other SCAFs, such as SCAF1, SCAF11, and PHRF1 (originally identified as SCAF9), elongation factors such as RPRD1A/B, RECQL5, and SPT6 (also known as SUPTH6), and the PAF complex. Additionally, we found 3′ end processing factors preferentially enriched in SCAF4 ELCAP-MS, such as CPSF factors. This fits with what we know about the function of SCAFs as mRNA transcript anti-terminators at intronic polyA sites and with their individual roles in regulation at the 3′ end of genes. The role of these proteins in controlling the elongation-termination transition is further supported by the fact that both proteins preferentially interact with a subset of transcriptionally engaged RNAPII complexes that are concomitantly bound by elongation factors as well as 3′ end processing factors but are depleted for splicing factors. This again fits nicely with what we know about the SCAF4 and SCAF8 function: SCAF4 and SCAF8 double KOs has little impact on RNAPII elongation rates or RNAPII termination past the distal polyA (pA) site but results specifically in an up-regulation of shorter mRNA transcript isoforms due to a failure to suppress premature termination.[1]

Using SCAF4 and SCAF8 as proof of principle, we thus conclude that ELCAP efficiently enables elongation-factor-specific RNAPII binding profiles genome-wide and, at the same time, provides the basis for proteomic investigation of RNAPII elongation complexes. We envision that the use of this method will provide important insights for other RNAPII-associated factors with a poorly characterized function.

Limitations of the study

In principle, ELCAP can be applied to any protein of interest that interacts with RNAPII. Here, we used an antibody that recognizes the phosphorylated CTD of RNAPII (4H8) for the second step of the purification of transcriptionally engaged RNAPII complexes. This is particularly well suited to study the binding pattern of transcription regulators/elongation factors that interact with elongating RNAPII throughout the gene body. However, for factors that primarily interact with the unphosphorylated RNAPII or only display weak binding toward the transcriptionally engaged RNAPII, the second step of RNAPII purification could be performed using antibodies against total RNAPII (such as D8L4Y), the unphosphorylated RNAPII (8WG16), or specific phosphorylated RNAPII-CTD forms. Due to the double-affinity purification, it is not possible to add spike ins prior to the two IP steps; we therefore use density scaling to compare differential binding patterns relative to the RNAPII single IP reference and the factor-specific RNAPII-binding profiles obtained from the double-IP procedure.

STAR★METHODS

Detailed methods are provided in the online version of this paper and include the following:

- KEY RESOURCES TABLE
- RESOURCE AVAILABILITY
  - Lead contact
  - Materials availability
  - Data and code availability
- EXPERIMENTAL MODEL AND SUBJECT DETAILS
  - Cell lines and culture conditions
  - Generation of stable cell lines
- METHOD DETAILS
  - Western blotting
  - Quantitative PCR (qPCR)
  - ELCAP for sequencing and mass spectrometry

- ELCAP-seq and mNET-seq analysis
- mNET-seq chromatin extraction
- QUANTIFICATION AND STATISTICAL ANALYSIS

SUPPLEMENTAL INFORMATION

Supplemental information can be found online at https://doi.org/10.1016/j.crmeth.2022.100368.

ACKNOWLEDGMENTS

We thank members of Francis Crick Institute's Proteomics and Advanced Sequencing Facilities for expert technical assistance and Barbara Dirac-Svejstrup for feedback on the manuscript. This work was supported by grants to J.Q.S. from the European Research Council (ERC Agreement 693327), a Laureate grant from the Novo Nordisk Foundation (NNF19OC0055875), and a Chair grant from the Danish National Research Foundation (DNRF153) and by the Francis Crick Institute (which receives its core funding from Cancer Research UK [FC001166], the UK Medical Research Council [FC001166], and the Wellcome Trust [FC001166]). L.H.G. was supported by the EMBO-LTF program (EMBO ALTF 1026-2014), a Hallas-Møller emerging investigator grant from the Novo Nordisk Foundation (NNF20OC0059959), and a Sapere Aude research leader program grant from Independent Research Fund Denmark (0165-00092B).

AUTHOR CONTRIBUTIONS

L.H.G. and J.Q.S. conceived the project. L.H.G. performed all experiments. L.H.G and R.M. performed the bioinformatic analysis. L.H.G. and J.Q.S. wrote the manuscript.

DECLARATION OF INTERESTS

The authors declare no competing interests.

Received: August 22, 2022
Revised: October 25, 2022
Accepted: November 18, 2022
Published: December 9, 2022

REFERENCES

1. Gregersen, L.H., Mitter, R., Ugalde, A.P., Nojima, T., Proudfoot, N.J., Agami, R., Stewart, A., and Svejstrup, J.Q. (2019). SCAF4 and SCAF8, mRNA anti-terminator proteins. Cell *177*, 1797–1813.e18. https://doi.org/10.1016/j.cell.2019.04.038.
2. Zatreanu, D., Han, Z., Mitter, R., Tumini, E., Williams, H., Gregersen, L., Dirac-Svejstrup, A.B., Roma, S., Stewart, A., Aguilera, A., and Svejstrup, J.Q. (2019). Elongation factor TFIIS prevents transcription stress and R-loop accumulation to maintain genome stability. Mol. Cell *76*, 57–69.e9. https://doi.org/10.1016/j.molcel.2019.07.037.
3. Corden, J.L. (2013). RNA polymerase II C-terminal domain: tethering transcription to transcript and template. Chem. Rev. *113*, 8423–8455. https://doi.org/10.1021/cr400158h.
4. Eick, D., and Geyer, M. (2013). The RNA polymerase II carboxy-terminal domain (CTD) code. Chem. Rev. *113*, 8456–8490. https://doi.org/10.1021/cr400071f.
5. Harlen, K.M., and Churchman, L.S. (2017). The code and beyond: transcription regulation by the RNA polymerase II carboxy-terminal domain. Nat. Rev. Mol. Cell Biol. *18*, 263–273. https://doi.org/10.1038/nrm.2017.10.
6. Jonkers, I., and Lis, J.T. (2015). Getting up to speed with transcription elongation by RNA polymerase II. Nat. Rev. Mol. Cell Biol. *16*, 167–177. https://doi.org/10.1038/nrm3953.
7. Noe Gonzalez, M., Blears, D., and Svejstrup, J.Q. (2021). Causes and consequences of RNA polymerase II stalling during transcript elongation. Nat. Rev. Mol. Cell Biol. *22*, 3–21. https://doi.org/10.1038/s41580-020-00308-8.
8. Mayer, A., Heidemann, M., Lidschreiber, M., Schreieck, A., Sun, M., Hintermair, C., Kremmer, E., Eick, D., and Cramer, P. (2012). CTD tyrosine phosphorylation impairs termination factor recruitment to RNA polymerase II. Science *336*, 1723–1725. https://doi.org/10.1126/science.1219651.
9. Hintermair, C., Heidemann, M., Koch, F., Descostes, N., Gut, M., Gut, I., Fenouil, R., Ferrier, P., Flatley, A., Kremmer, E., et al. (2012). Threonine-4 of mammalian RNA polymerase II CTD is targeted by Polo-like kinase 3 and required for transcriptional elongation. EMBO J. *31*, 2784–2797. https://doi.org/10.1038/emboj.2012.123.
10. Heidemann, M., Hintermair, C., Voß, K., and Eick, D. (2013). Dynamic phosphorylation patterns of RNA polymerase II CTD during transcription. Biochim. Biophys. Acta *1829*, 55–62. https://doi.org/10.1016/j.bbagrm.2012.08.013.
11. Komarnitsky, P., Cho, E.J., and Buratowski, S. (2000). Different phosphorylated forms of RNA polymerase II and associated mRNA processing factors during transcription. Genes Dev. *14*, 2452–2460. https://doi.org/10.1101/gad.824700.
12. Schwalb, B., Michel, M., Zacher, B., Frühauf, K., Demel, C., Tresch, A., Gagneur, J., and Cramer, P. (2016). TT-seq maps the human transient transcriptome. Science *352*, 1225–1228. https://doi.org/10.1126/science.aad9841.
13. Gregersen, L.H., Mitter, R., and Svejstrup, J.Q. (2020). Using TTchem-seq for profiling nascent transcription and measuring transcript elongation. Nat. Protoc. *15*, 604–627. https://doi.org/10.1038/s41596-019-0262-3.
14. Mayer, A., di Iulio, J., Maleri, S., Eser, U., Vierstra, J., Reynolds, A., Sandstrom, R., Stamatoyannopoulos, J.A., and Churchman, L.S. (2015). Native elongating transcript sequencing reveals human transcriptional activity at nucleotide resolution. Cell *161*, 541–554. https://doi.org/10.1016/j.cell.2015.03.010.
15. Nojima, T., Rebelo, K., Gomes, T., Grosso, A.R., Proudfoot, N.J., and Carmo-Fonseca, M. (2018). RNA polymerase II phosphorylated on CTD serine 5 interacts with the spliceosome during Co-transcriptional splicing. Mol. Cell *72*, 369–379.e4. https://doi.org/10.1016/j.molcel.2018.09.004.
16. Schlackow, M., Nojima, T., Gomes, T., Dhir, A., Carmo-Fonseca, M., and Proudfoot, N.J. (2017). Distinctive patterns of transcription and RNA processing for human lincRNAs. Mol. Cell *65*, 25–38. https://doi.org/10.1016/j.molcel.2016.11.029.
17. Nojima, T., Gomes, T., Grosso, A.R.F., Kimura, H., Dye, M.J., Dhir, S., Carmo-Fonseca, M., and Proudfoot, N.J. (2015). Mammalian NET-seq reveals genome-wide nascent transcription coupled to RNA processing. Cell *161*, 526–540. https://doi.org/10.1016/j.cell.2015.03.027.
18. Bentley, D.L. (2014). Coupling mRNA processing with transcription in time and space. Nat. Rev. Genet. *15*, 163–175. https://doi.org/10.1038/nrg3662.
19. Buratowski, S. (2009). Progression through the RNA polymerase II CTD cycle. Mol. Cell *36*, 541–546. https://doi.org/10.1016/j.molcel.2009.10.019.
20. Aygün, O., Svejstrup, J., and Liu, Y. (2008). A RECQ5-RNA polymerase II association identified by targeted proteomic analysis of human chromatin. Proc. Natl. Acad. Sci. USA *105*, 8580–8584. https://doi.org/10.1073/pnas.0804424105.
21. Boeing, S., Williamson, L., Encheva, V., Gori, I., Saunders, R.E., Instrell, R., Aygün, O., Rodriguez-Martinez, M., Weems, J.C., Kelly, G.P., et al. (2016). Multiomic analysis of the UV-induced DNA damage response. Cell Rep. *15*, 1597–1610. https://doi.org/10.1016/j.celrep.2016.04.047.
22. Nojima, T., Gomes, T., Carmo-Fonseca, M., and Proudfoot, N.J. (2016). Mammalian NET-seq analysis defines nascent RNA profiles and associated RNA processing genome-wide. Nat. Protoc. *11*, 413–428. https://doi.org/10.1038/nprot.2016.012.
23. Ehrensberger, A.H., Kelly, G.P., and Svejstrup, J.Q. (2013). Mechanistic interpretation of promoter-proximal peaks and RNAPII density maps. Cell *154*, 713–715. https://doi.org/10.1016/j.cell.2013.07.032.

CellPress
OPEN ACCESS

24. Fong, N., Brannan, K., Erickson, B., Kim, H., Cortazar, M.A., Sheridan, R.M., Nguyen, T., Karp, S., and Bentley, D.L. (2015). Effects of transcription elongation rate and Xrn2 exonuclease activity on RNA polymerase II termination suggest widespread kinetic competition. Mol. Cell *60*, 256–267. https://doi.org/10.1016/j.molcel.2015.09.026.

25. Fischl, H., Howe, F.S., Furger, A., and Mellor, J. (2017). Paf1 has distinct roles in transcription elongation and differential transcript fate. Mol. Cell *65*, 685–698.e8. https://doi.org/10.1016/j.molcel.2017.01.006.

26. Uzun, Ü., Brown, T., Fischl, H., Angel, A., and Mellor, J. (2021). Spt4 facilitates the movement of RNA polymerase II through the +2 nucleosomal barrier. Cell Rep. *36*, 109755. https://doi.org/10.1016/j.celrep.2021.109755.

27. Mylonas, C., and Tessarz, P. (2019). NET-prism enables RNA polymerase-dedicated transcriptional interrogation at nucleotide resolution. RNA Biol. *16*, 1156–1165. https://doi.org/10.1080/15476286.2019.1621625.

28. Ramírez, F., Ryan, D.P., Grüning, B., Bhardwaj, V., Kilpert, F., Richter, A.S., Heyne, S., Dündar, F., and Manke, T. (2016). deepTools2: a next generation web server for deep-sequencing data analysis. Nucleic Acids Res. *44*, W160–W165. https://doi.org/10.1093/nar/gkw257.

29. Tyanova, S., Temu, T., and Cox, J. (2016). The MaxQuant computational platform for mass spectrometry-based shotgun proteomics. Nat. Protoc. *11*, 2301–2319. https://doi.org/10.1038/nprot.2016.136.

30. Martin, M. (2011). Cutadapt removes adapter sequences from high-throughput sequencing reads. EMBnet. J. *17*, 10–12. https://doi.org/10.14806/ej.17.1.200.

31. Kim, D., Langmead, B., and Salzberg, S.L. (2015). HISAT: a fast spliced aligner with low memory requirements. Nat. Methods *12*, 357–360. https://doi.org/10.1038/nmeth.3317.

32. Li, H., Handsaker, B., Wysoker, A., Fennell, T., Ruan, J., Homer, N., Marth, G., Abecasis, G., and Durbin, R.; 1000 Genome Project Data Processing Subgroup (2009). The sequence alignment/map format and SAMtools. Bioinformatics *25*, 2078–2079. https://doi.org/10.1093/bioinformatics/btp352.

33. Quinlan, A.R., and Hall, I.M. (2010). BEDTools: a flexible suite of utilities for comparing genomic features. Bioinformatics *26*, 841–842. https://doi.org/10.1093/bioinformatics/btq033.

34. Kent, W.J., Zweig, A.S., Barber, G., Hinrichs, A.S., and Karolchik, D. (2010). BigWig and BigBed: enabling browsing of large distributed datasets. Bioinformatics *26*, 2204–2207. https://doi.org/10.1093/bioinformatics/btq351.

STAR★METHODS

KEY RESOURCES TABLE

REAGENT or RESOURCE	SOURCE	IDENTIFIER
Antibodies		
SCAF4	Bethyl	RRID: AB_2620300
SCAF8	Bethyl	RRID: AB_2253436
RNAPII 4H8	The Francis Crick Institute	N/A
RNAPII 3E10	kind gift from Dirk Eick	N/A
RNAPII 3E8	kind gift from Dirk Eick	N/A
FLAG 1804	Merck	RRID: AB_262044
Chemicals, peptides, and recombinant proteins		
Benzonase	Merck Millipore	70746-4
anti-FLAG M2 Affinity Gel	Merck	A2220
Dynabeads Protein G	Thermo	10004D
iTaq Universal SYBR Green Supermix	BioRad	172-5124
Critical commercial assays		
Quick-RNA MicroPrep	Zymo	R1050
Qubit/RNA HS assay kit	Thermo	Q32852
NEBNext Multiplex Small RNA Library Prep Set for Illumina	NEB	E7300
Qubit/HS dsDNA assay kit	Thermo	Q32851
RNeasy kit	Qiagen	74104
TaqMan Reverse Transcription Reagents	Thermo	N8080234
Deposited data		
ELCAP-seq data	This paper	GEO: GSE207568
RNAPII mNET-seq	Gregersen et al. 2019[1]	GEO: GSE121826
RNAPII 4H8 ChIP-seq	Zatreanu et al., 2019[28]	GEO: GSE132400
Experimental models: Cell lines		
Flp-In T-REx HEK293 cells	Thermo Fisher Scientific	R78007
FLAG-SCAF4 Flp-In T-REx HEK293 cell line	Gregersen et al. 2019[1]	N/A
FLAG-SCAF8 Flp-In T-REx HEK293 cell line	Gregersen et al. 2019[1]	N/A
Oligonucleotides		
All oligonucleotides used in this study are listed in Table S2	This paper	N/A
Recombinant DNA		
pFRT/TO/SCAF4-FLAG	Gregersen et al. 2019[1]	Addgene: 122469
pFRT/TO/FLAGHA-SCAF8	Gregersen et al. 2019[1]	Addgene: 122470
Software and algorithms		
MaxQuant v1.3.05	Tyanova et al., 2016[22]	https://www.maxquant.org/
Perseus software v1.4.0.11	Tyanova et al., 2016[22]	https://maxquant.net/perseus/
TrimGalore v0.4.4	Martin 2011[2]	https://github.com/FelixKrueger/TrimGalore
HISAT2 v2.0.4	Kim et al., 2011[29]	http://daehwankimlab.github.io/hisat2/
SAMtools	Handsaker et al., 2009[30]	http://www.htslib.org/
BEDtools	Quinlan and Hall 2010[31]	https://bedtools.readthedocs.io/en/latest/
bedGraphToBigWig	Kent et al., 2010[32]	https://www.encodeproject.org/software/bedgraphtobigwig/

RESOURCE AVAILABILITY

Lead contact

Further information and requests for resources and reagents should be directed to and will be fulfilled by the Lead Contact, Jesper Q. Svejstrup (jsvejstrup@sund.ku.dk).

Materials availability

Plasmids used in this study have been deposited in Addgene. Catalog numbers are listed in the key resources table.

Data and code availability

- ELCAP sequencing data generated as part of this study is available under GEO number GSE207568. Total RNAPII mNET-seq[1] data is available through GEO: GSE121826. RNAPII 4H8 ChIP-seq[2] data is available through GEO: GSE132400.
- This paper does not report original code.
- Any additional information required to reanalyze the data reported in this paper is available from the lead contact upon request.

EXPERIMENTAL MODEL AND SUBJECT DETAILS

Cell lines and culture conditions

Flp-In T-REx HEK293 cells (R78007, Thermo Fisher Scientific, human embryonic kidney epithelial, female origin) were cultured in high glucose DMEM (11965118, Thermo Fisher Scientific) supplemented with 10% v/v FBS, 100 U/mL penicillin, 100 μg/mL streptomycin, 2 mM L-glutamine, 100 μg/mL zeocin and 15 μg/mL blasticidin at 37°C with 5% CO2 and routinely passaged 2–3 times a week. All cell lines were confirmed to be mycoplasma-free.

Generation of stable cell lines

SCAF4 and SCAF8 ORF plasmids and Flp-In T-REx HEK293 cell lines expressing stably expressing FLAG-tagged SCAF4 or SCAF8 are described previously.[1]

METHOD DETAILS

Western blotting

Protein extracts from cell fractionations or IPs were separated on 3–8% Tris-Acetate (3450130, BioRad) or 4–15% TGX gels (56711084 or 56711085, BioRad) and transferred to nitrocellulose membranes (10600002, GE Healthcare Life Sciences). Membranes were blocked in 5% (w/v) skimmed milk in PBS-T (PBS, 0.1% (v/v) Tween 20) for 1 h at room temperature and incubated with primary antibody (in 5% (w/v) skimmed milk in PBS-T) overnight at 4°C. Primary antibodies used were SCAF4 (A303-951A, Bethyl, RRID: AB_2620300), SCAF8 (A301-037A, Bethyl, RRID: AB_2253436), RNAPII 4H8 (mouse monoclonal 4H8 raised against the phosphorylated RNAPII CTD, Cell services, The Francis Crick Institute), RNAPII 3E10 (rat monoclonal to Ser2P RNAPII (3E10), kind gift from Dirk Eick), RNAPII 3E8 (rat monoclonal to Ser5P RNAPII (3E8), kind gift from Dirk Eick), FLAG (F1804, Merck RRID: AB_262044). Membranes were washed several times in PBS-T, incubated with HRP-conjugated secondary antibody (sc-516102, anti-mouse HRP Santa Cruz, 711-035-152, anti-rabbit HRP Jackson Immuno Research, or 112-035-003, anti-rat Jackson Immuno Research) in 5% (w/v) skimmed milk in PBS-T and visualised using SuperSignal™ West Pico PLUS or Dura Chemiluminescent Substrate ECL reagent (34577 and 34075, Thermo Fisher Scientific).

Quantitative PCR (qPCR)

Total RNA was extracted using the RNeasy kit (QIAGEN, 74104) following the instructions of the manufacturer including an on-column DNase treatment (QIAGEN, 79,254). Reverse transcription was performed using TaqMan Reverse Transcription Reagents (Thermo Fisher Scientific, N8080234) using random hexamers. cDNA was amplified using iTaq Universal SYBR Green Supermix (BioRad, 172-5124) with 30 cycles of 15 s denaturation at 94°C, 15 s annealing at 60°C, and 20 s extensions at 72°C. Primers amplifying mature GAPDH were used as normalization control. Primer sequences are listed in Table S2.

ELCAP for sequencing and mass spectrometry

Cellular fractionation

Flp-In T-REx HEK293 cells and Flp-In T-REx HEK293 stably expressing Dox-inducible FLAG-SCAF4 or FLAG-SCAF8 (induced overnight by the addition of 1 μg/mL doxycycline) were used for cellular fractionation. Cells were harvested by scraping in ice-cold PBS, washed once in cold PBS, and pelleted by centrifugation at 1,500 rpm for 5 min at 4°C. Cells were then fractionated to obtain a soluble fraction (containing cytosolic and nucleoplasmic proteins) and a chromatin fraction. All buffers were pre-cooled on ice and samples kept on ice all the time during the cell fractionation. Phosphatase inhibitors (PhosSTOP™, 04906837001, Merck) and Protease Inhibitor Cocktail (05056489001, Merck) were added fresh to all buffers. Firstly, cells were resuspended in 2 pellet volumes of hypotonic

buffer (10 mM HEPES pH 7.5, 10 mM KCl, 1.5 mM $MgCl_2$, 20 mM NEM (E3876, N-ethylmaleimide, Merck), incubated on ice for 15 min and dounce homogenized with 20 strokes using a loose pestle. Nuclei were pelleted at 3,900 rpm for 15 min and supernatant collected as cytoplasmic fraction. The remaining pellet was resuspended in 2 pellet volumes (original cell pellet volumes) nucleoplasmic extraction buffer (20 mM HEPES pH 7.9, 1.5 mM $MgCl_2$, 150 mM potassium acetate, 10 % (v/v) glycerol and 0.05 % (v/v) NP-40), incubated on ice for 20 min and cleared by centrifugation at 20,000g for 20 min at 4°C. Supernatant was collected as nucleoplasmic fraction. After correcting the cytoplasmic fractions to 10% (v/v) glycerol, 3 mM EDTA, 0.05% (v/v) NP-40 and 150 mM NaCl final concentration, the cytoplasmic and nucleoplasmic fraction were pooled to obtain a combined soluble fraction. The remaining pellet was resuspended in chromatin digestion buffer (20 mM HEPES pH 7.9, 1.5 mM $MgCl_2$, 10% (v/v) glycerol, 150 mM NaCl, 0.1% (v/v) NP-40 and 250 U/mL Benzonase (Merck Millipore, 70,746-4)) and incubated for 1 h at 4°C. Benzonase digested samples were centrifuged at 20,000g for 20 min at 4°C and supernatant collected as chromatin fraction.

Immunoprecipitations

FLAG immunoprecipitations were carried out using anti-FLAG M2 Affinity Gel (A2220, Merck). 3 mL of chromatin extracts were incubated with 200 uL bead slurry at 4°C for 1.5 h. Beads were washed 4 times 5mL of IP wash buffer (150 mM NaCl, 20 mM Tris-HCl pH 7.5, 1.5 mM $MgCl_2$, 3 mM EDTA, 10% (v/v) glycerol, 0.1% (v/v) NP-40, phosphatase inhibitors (PhosSTOP, 04906837001, Merck) and protease inhibitor cocktail, 05056489001, Merck)), followed by two washes on a spin column (Thermo Fisher Scientific, 69705) with 200 uL IP wash buffer per wash. FLAG elutions were carried out on the spin column by addition of a stopper to the bottom of the spin column followed by addition of 300 uL 1 mg/mL 3xFLAG peptide (Peptide Chemistry, The Francis Crick Institute) dissolved in IP wash buffer. Beads were incubated with FLAG-peptide elution buffer for 1 h at 4°C. 5% of the FLAG elutions were run on an SDS-PAGE for western blot to confirm immunoprecipitation of full-length SCAF4 and SCAF8. The remaining FLAG elutions were diluted to 1 mL per sample by addition of IP wash buffer and used for the subsequent RNAPII immunoprecipitation. Transcriptionally engaged phosphorylated RNAPII complexes were immunoprecipitated using monoclonal RNAPII 4H8 antibody conjugated to Dynabeads Protein G (10004D, Thermo). 50 uL Dynabeads Protein G per sample were washed 3 times in PBS, 0.05% NP-40 and incubated with 5 ug of RNAPII 4H8 in a total volume of 1.2 mL for 2 h at room temperature. 4H8 conjugated beads were washed 3 times in PBS, 0.05% NP-40, resuspended in 100 uL PBS, 0.05% NP-40 and added to the 1 mL samples containing the FLAG elution from the first IP step (double IP). For the single RNAPII IPs 4H8 conjugated beads were added direct to chromatin extracts prepared as described above. Samples were incubated 2 h at 4°C on a rotating wheel. Dynabeads were then washed 5 times in IP wash buffer using a magnetic stand. After the final wash step, 5% of the beads were removed for a western blot control of the immunoprecipitation.

Preparation of RNA for sequencing (ELCAP-seq)

The remaining beads were used directly for RNA extraction by addition of 300 uL RNA extraction mix: consisting of 100 uL IP wash buffer +100 uL Zymo RNA lysis buffer (R1050, Zymo Research Quick-RNA Micro-Prep) + 100 uL 100% ethanol) directly to the dry beads. Beads were incubated 2 min with the RNA extraction mix at room temperature and placed back on the magnetic stand. Supernatant containing the RNA was transferred to a new tube and used for isolation of both small (17-200nt RNA) and >200nt RNA using the Zymo Research Quick-RNA Micro-Prep (R1050) accordingly to the manufacturer's instructions. Finally, the purified RNA was eluted in 15 uL RNase-free water. 3 uL of purified RNA was used for a bioanalyzer control (2100 Bioanalyzer Agilent). RNA concentrations were measured using a Qubit/RNA HS assay (Q32852, Thermo). For the single step RNAPII immunoprecipitations 50 uL Dynabeads Protein G (10004D, Thermo) per sample were washed 3 times in PBS, 0.05% NP-40 and incubated with 5 ug of RNAPII 4H8 in a total volume of 1.2 mL for 2 h at room temperature. 4H8 conjugated beads were washed 3 times in PBS, 0.05% NP-40. 4H8 conjugated beads were resuspended in 100 uL and added to 3mL of chromatin extracts. Samples were washed 5 times in IP wash buffer using a magnetic stand and 5% of the beads removed for a western blot control of the immunoprecipitation. The remaining beads were used directly for RNA extraction by addition of 300 uL RNA extraction mix and RNA extracted as described above for the double affinity purification. Small RNA libraries were prepared using NEBNext Multiplex Small RNA Library Prep Set for Illumina (E7300, NEB). There is no need for end-repair of the RNA prior to the library prep as the Benzonase generated ends are compatible with adapter ligations. The PCR amplified libraries were amplified with 12 cycles and products with the size range of 140-230 bp (corresponding to an insert size range of 20-90 nt) and gel purified using a 6% Novex TBE gel (EC6265BOX, Thermo). Gel slices were crushed using RNase-free single-use pellet pestles (12-141-364, Fisher Scientific) and incubated in 250 uL gel elution buffer (supplied with NEBNext kit) for 2 h at room temperature. Gel pieces were transferred to a Spin-X gel filtration column (CLS8160, Merck) and centrifuged for 2 min at 13.000rpm. Flow-through was collected and DNA precipitated by the addition of 750uL 100% ethanol, 25 uL 3M sodium acetate pH = 5.5 and 1 uL linear acrylamide overnight at −20°C followed by centrifugation at 13.000rpm for 30 min at 4°C. Pellets were washed in 80% ethanol, dried and resuspended in 10 uL TE buffer. DNA concentration of the PCR library was measured by Qubit/HS dsDNA kit (Q32851, Thermo). Library QC to confirm size distribution was performed on an Agilent 4200 TapeStation. Samples were sequenced on a HiSeq4000 (Illumina) (SE75 run).

Proteomics of RNAPII complexes (ELCAP-MS)

For mass spectrometry of immunoprecipitations were carried out as described above and proteins were eluted from beads by glycine elution instead of being used for RNA extractions. 50 uL glycine elution buffer (100 mM glycine pH 2.4) was added directly to dry beads, incubated 5 min at room temperature and vortexed. Afterward supernatant (eluted proteins) were transferred to a new tube and neutralised by addition of 25 uL 1 M Tris pH 8.8. An equal volume of 2x SDS containing loading buffer was added and samples subjected to SDS-PAGE. Samples were migrated 2 cm into the gel and excised. Proteins were in-gel digested with trypsin, using a Janus Automated Workstation (Perkin Elmer), and peptides were analyzed using an LTQ Orbitrap-Velos mass spectrometer

coupled to an Ultimate3000 HPLC equipped with an EASY-Spray nanosource (Thermo Fisher Scientific). Raw data was processed using MaxQuant v1.3.05.[29] Due to several identical peptides between SCAF4 and SCAF8, the MaxQuant analysis was done separately for the SCAF4 and SCAF8 immuno-precipitates to avoid wrongly assigning common peptides, which would otherwise assign common peptides to the protein with the highest overall peptide count. The proteingroup.txt output table was imported into Perseus software v1.4.0.11[29] for further statistical processing, and visualization. Statistical parameters for volcano plots were calculated using two-sided t test for data from two biological replicates (each containing information form triplicate injections). To generate datasets containing merged quantifications for the two biological replicates, only peptides with a combined count >3 were considered. For volcano plots the log2 t test difference were plotted against -log2 t test p values. Proteins with a log2 t test difference >2 and -log2 t test p value > 2 were defined as enriched and termed RNAPII interactors.

ELCAP-seq and mNET-seq analysis

Data processing was adapted to deal both with single end ELCAP-seq data and previously published paired-end mNET-seq data.[17] Briefly, reads were adapter trimmed using TrimGalore v0.4.4[29]. Reads <10 bp in length and those with a maximum error rate >0.05 were discarded. HISAT2 v2.0.4 was used to align remaining reads against the GRCh38 genome build in a strand-specific manner, allowing for at most 5 distinct primary alignments for each read.[31] Reads were sorted and indexed using SAMtools[32] and multi-mapping reads were removed. Picard were used to remove duplicate reads and those not mapping in proper pairs (for the paired end mNET-seq data). For ELCAP-seq, stranded and un-stranded read-level bigwig files directly from the filtered single-end BAM files using BEDTools[33] to create bedgraph files that are in turn used to make bigwig files using bedGraphToBigWig[34] (assumes an FR read orientation, which is the case for all data). Bigwig files were created at maximum (i.e., single-bp, not binned) resolution. For the paired-end mNET-seq data, BAM files were split into 4 pieces (P1 forward, P2 forward, P1 reverse and P2 reverse), before merging of the two forward and two reverse components into distinct temporary files prior to bigwig creation. To obtain single nucleotide resolution (SNR) data (mapping of the last incorporated nucleotide by RNAPII), we extracted the 3' ends of unpaired reads, or the 5' ends of second-in-pair reads. For the paired-end mNET-seq data, the second-in-pair carried the opposite strand information to the first in pair, thus it was necessary to "flip" the resulting strand information on the resulting bigwig files. All of this was done using BEDTools/bedGraphToBigWig as above. For the purposes of visualisation, the generation of bigwig files was repeated from BAM files merged across biological triplicates to increase coverage depth.

Deeptools[28] were used to create strand-specific feature profiles and heatmaps directly from the SNR bp-resolution merged bigwig files. For all feature profiles (metagene, TES profiles and profiles around pA sites) we density scaled the merged ELCAP data. Density scaling was performed by normalising the raw count data so that the area under the curve for each individual sample is always 1. Then density scaling was performed after bin size selection. We found this to provide a robust comparison of binding behaviors for the single RNAPII IP refence and the RNAPII subpopulations (double IP). For this we used the Ensembl definition of protein-coding genes from standard chromosomes (1–22,X,Y), n = 19,919. Metagenes were defined +/− 5 kb, with the upstream 5 kb and downstream 5 kb regions split into 100 bp bins each. The gene-bodies were scaled to 15 kb and divided into 100 bp bins. TES profiles were similarly profiled +/− 5 kb and split into 100 bp bins.

For the ELCAP-seq read distribution around polyA site, we used 421 polyA sites for the distal polyA profiles and 621 for the proximal (intronic) polyA profiles. These were taken from the high confidence polyA site, that we identified previously from 3′end-seq in HEK293 Flp-In TREX cells.[1] They are unique sites from protein coding genes residing on chr 1–22/X/Y. For the density profiles, we used a bin size of 40 bp, which translates to 100 bins over the −/+2 kb region.

Readthrough ratios (coverage density expressed as reads-per-kilobase (RPK) in the 50 kb downstream of the TES relative to the coverage density in the last exon) were calculated for all protein-coding genes in all samples as reads. For all genes boxplots, all genes (protein-coding, standard chromosomes, one representative transcript per gene based on strongest support level, then transcript genomic width), n = 19,919. For readthrough genes in *SCAF4* KOs cells we used previously published SCAF4 readthrough genes, n = 1,281.[1]

mNET-seq chromatin extraction

To compare our ELCAP chromatin extraction with the mNET-seq protocol we carried out a parallel cellular fractionation. For both the ELCAP-seq and mNET-seq we started with 5 × 15cm dishes of cultured cells. The ELCAP extraction was carried out as described above and mNET-seq as previously described.[22] Briefly, cells were resuspended in cold HLB + N (10mM Tris-HCl (pH 7.5), 10 mM NaCl, 2.5 mM MgCl2 and 0.5% NP-40) and incubated for 5 min on ice. The sample was underlayered with HLB + NS (10 mM Tris-HCl pH 7.5, 10 mM NaCl, 2.5 mM MgCl2, 0.5% NP-40 and 10% sucrose) and centrifuged at 420g for 5 min at 4°C to pellet nuclei. The nuclei were lysed by addition of NUN1 buffer (20 mM Tris-HCl (pH 7.9), 75 mM NaCl, 0.5 mM EDTA and 50% glycerol), resuspended and transferred to a new tube, whereafter 10 times the volume of NUN2 buffer (20 mM HEPES-KOH (pH 7.6), 300 mM NaCl, 0.2 mM EDTA, 7.5 mM MgCl2, 1% NP-40 and 1 M urea) is added. Samples were incubated on ice for 15 min and centrifuged at 16,000g for 10 min to pellet chromatin. Chromatin was resuspended in Micrococcal nuclease reaction buffer (50 mM Tris-HCl, 5 mM CaCl2) supplement with 100 μg/mL Purified BSA (B9000S, NEB) and 40 gel unit/μL MNase (M0247, NEB). Chromatin was digested by incubation at 37°C for 90 s at 1,400 rpm on a thermomixer. MNase activity was inhibited by addition of EGTA and solubilised chromatin diluted 10X in NET-2 buffer (50 mM Tris-HCl (pH 7.4, 150 mM NaCl and 0.05% NP-40) prior to immunoprecipitations. For both the ELCAP

and mNET-seq extraction procedures, the entire sample was used for a single step RNAPII IP as described above. For the input, unbound and IP samples, an equal fraction of the samples were used for western blotting to allow direct comparison.

QUANTIFICATION AND STATISTICAL ANALYSIS

The significance between RNAPII ELCAP, SCAF4-RNAPII ELCAP and SCAF8-RNAPII ELCAP binding to genes (n = 1,281) affected by transcriptional readthrough in *SCAF4* KO cells were calculated Wilcoxon ranked t test. Ratios with an infinite value were dropped.

Cell Reports Methods, Volume *2*

Supplemental information

Elongation factor-specific capture of RNA polymerase II complexes

Lea H. Gregersen, Richard Mitter, and Jesper Q. Svejstrup

Figure S1. Establishment of ELCAP, related to Figure 1

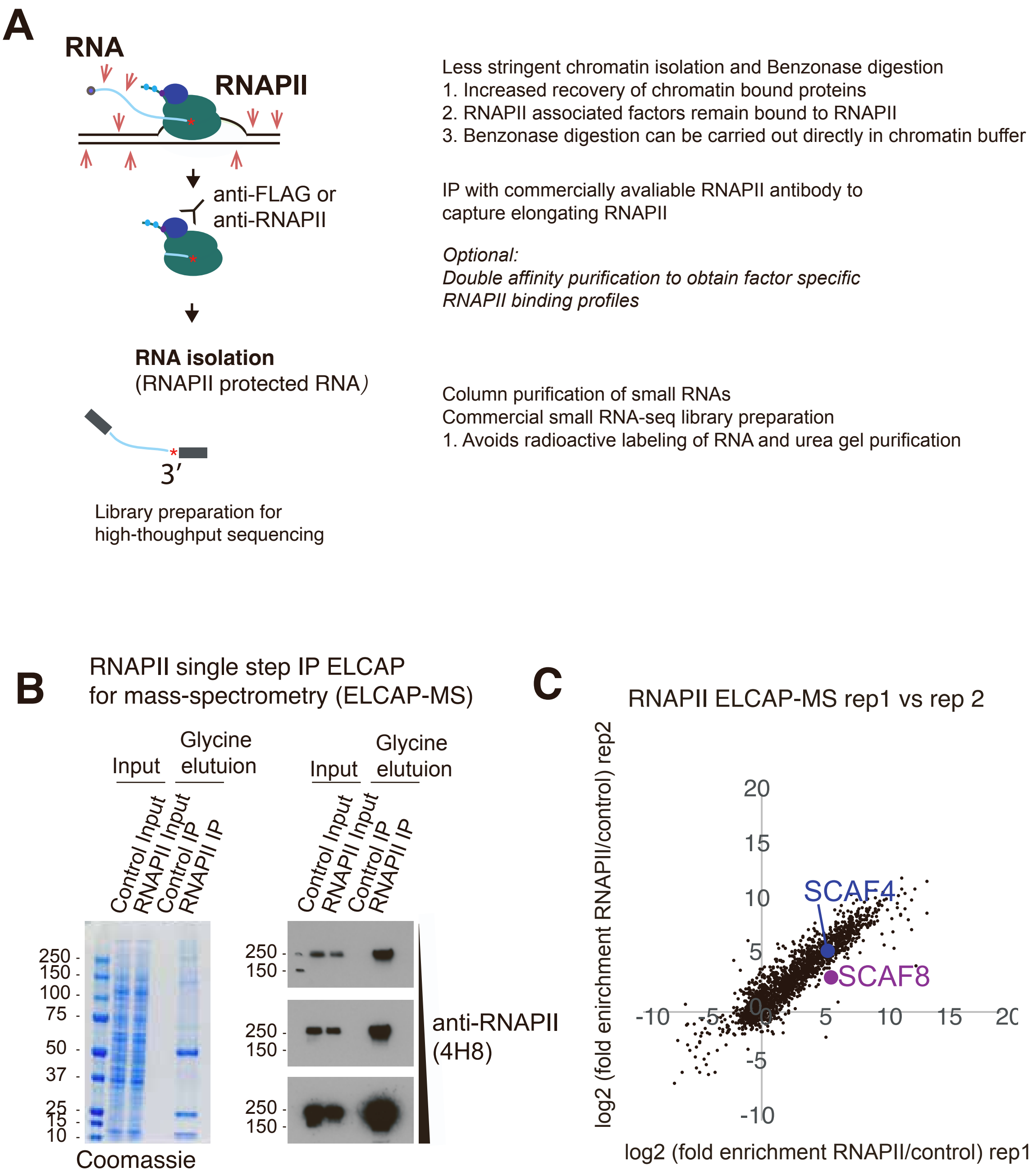

Figure S2. Comparison of RNAPII ELCAP-MS and RNAPII mNET-MS, related to Figure 2 and 3

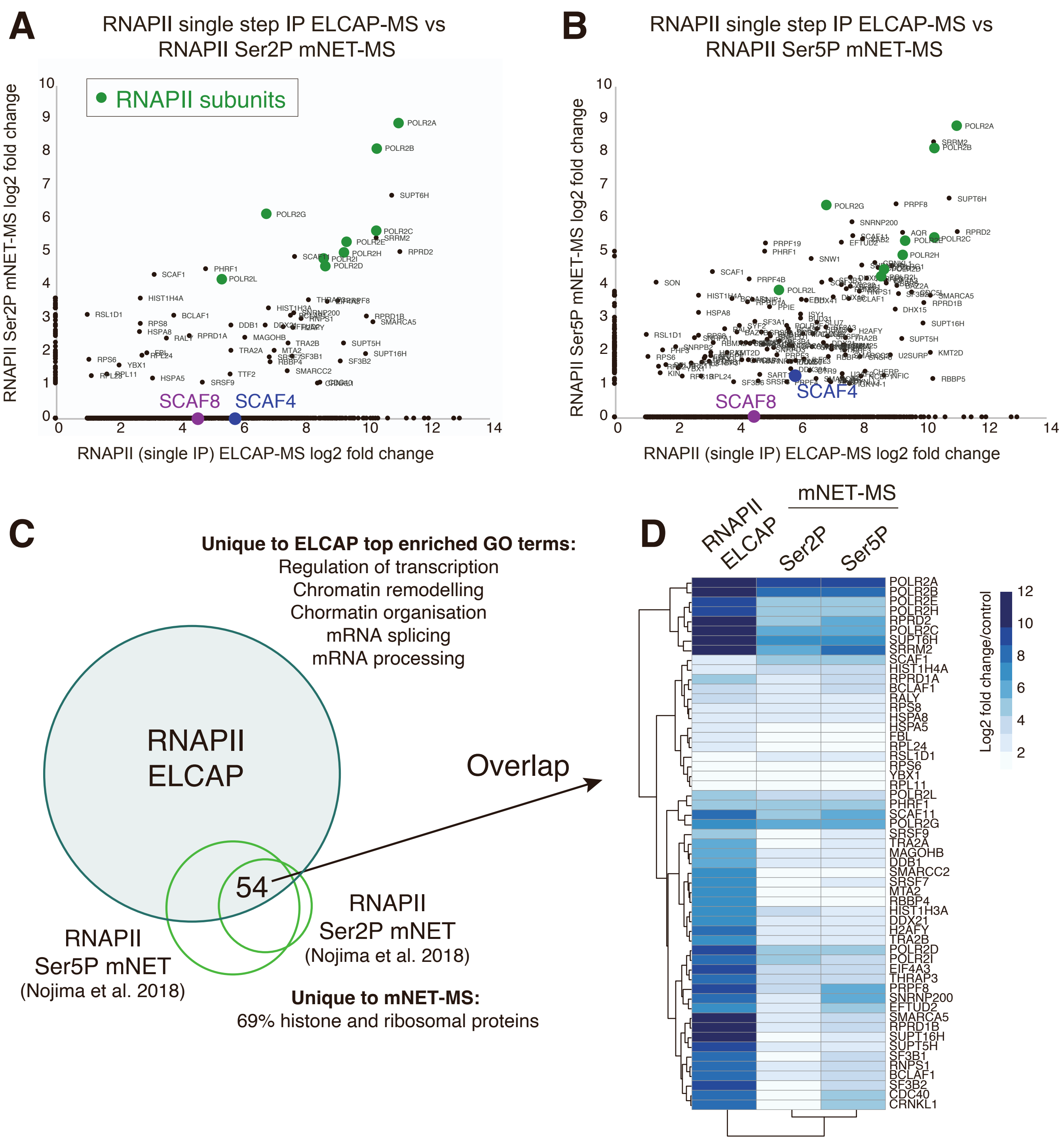

Figure S3. Single and double ELCAP purification of RNAPII, SCAF4- and SCAF8-RNAPII, related to Figure 4 and 5

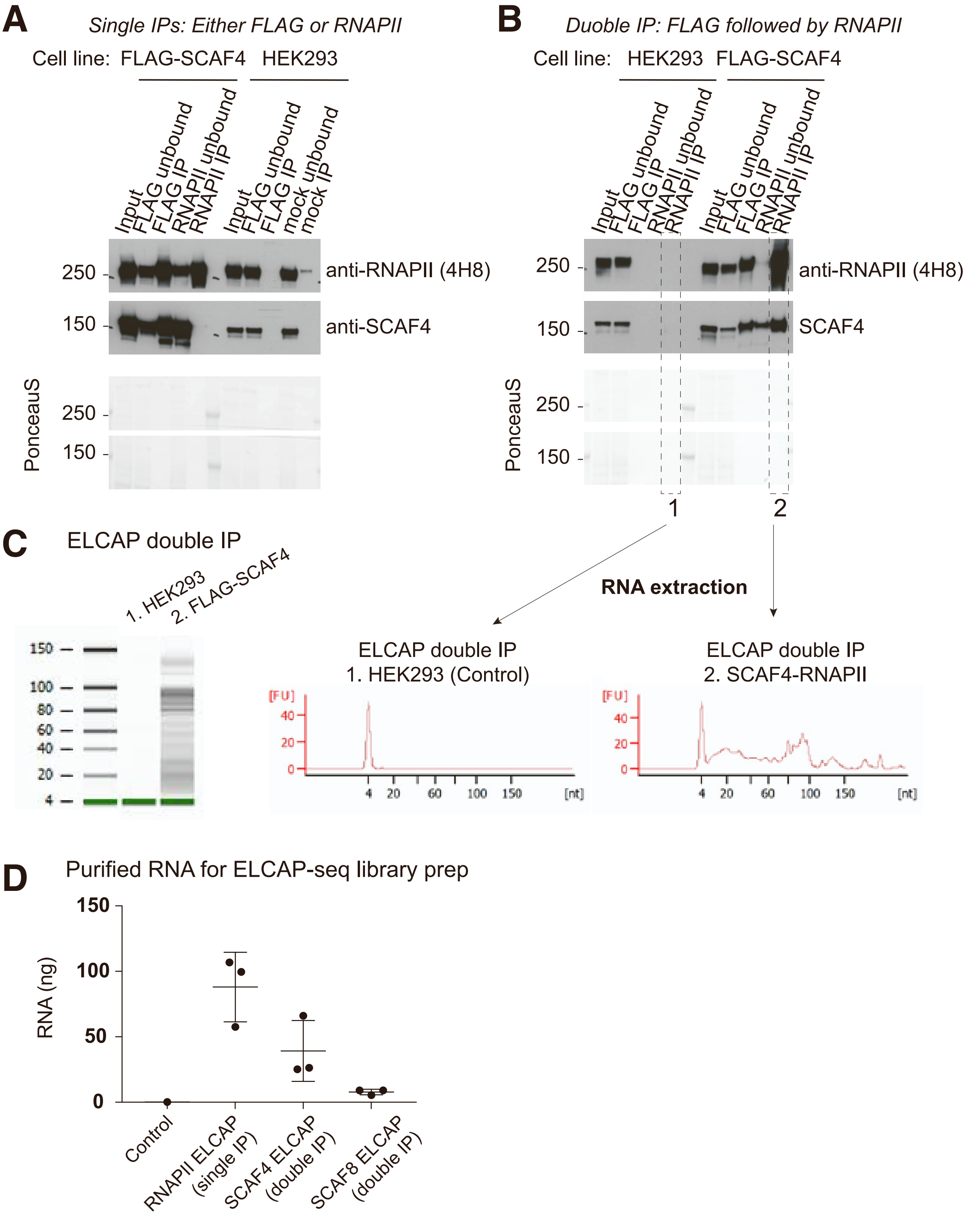

Figure S4. RNAPII ELCAP-seq profiles compared to ChIP-seq data, related to Figure 4

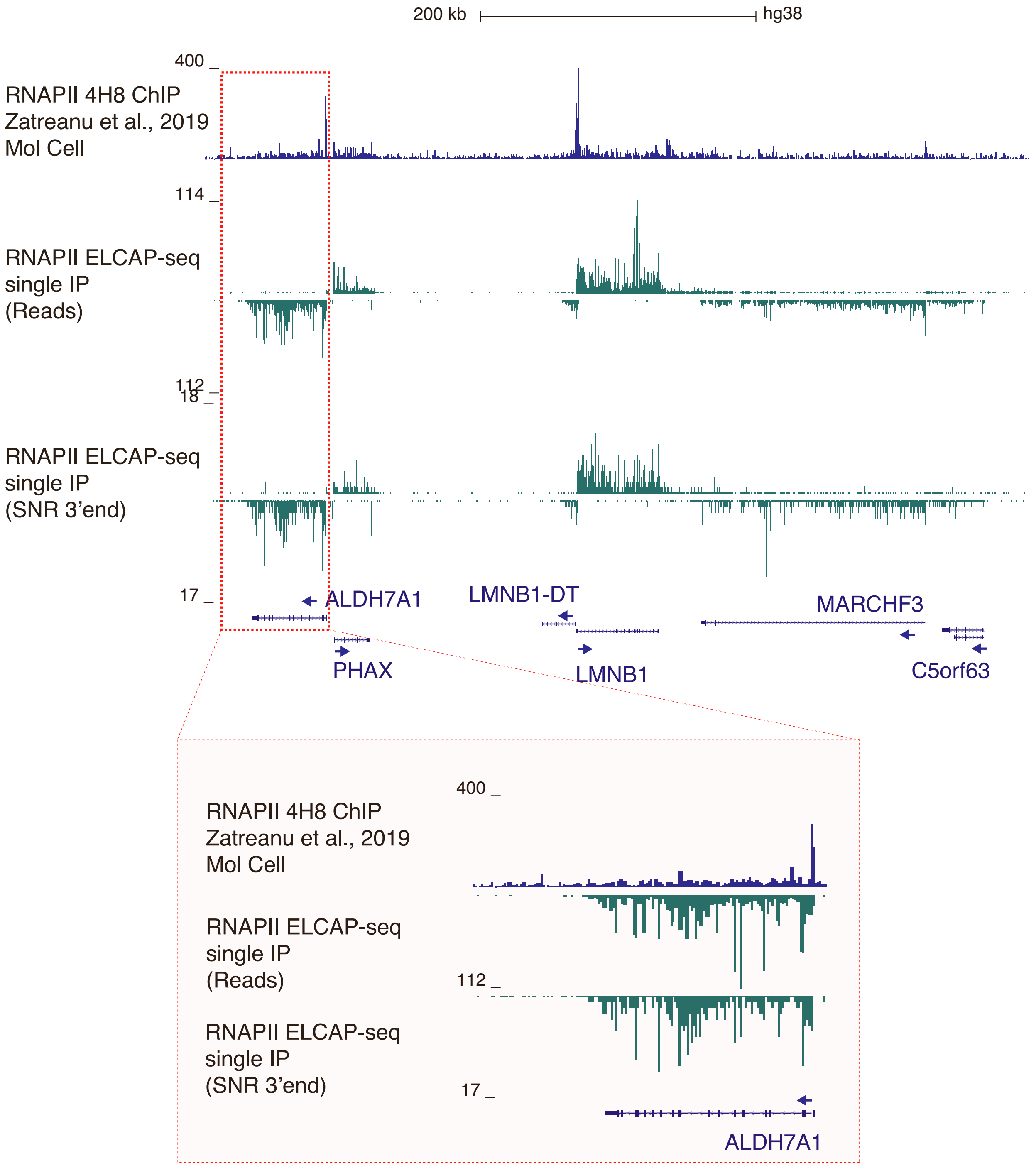

Figure S5. Transcriptionally engaged RNAPII, SCAF4-RNAPII and SCAF8-RNAPII ELCAP-seq profiles, related to Figure 5 and 6

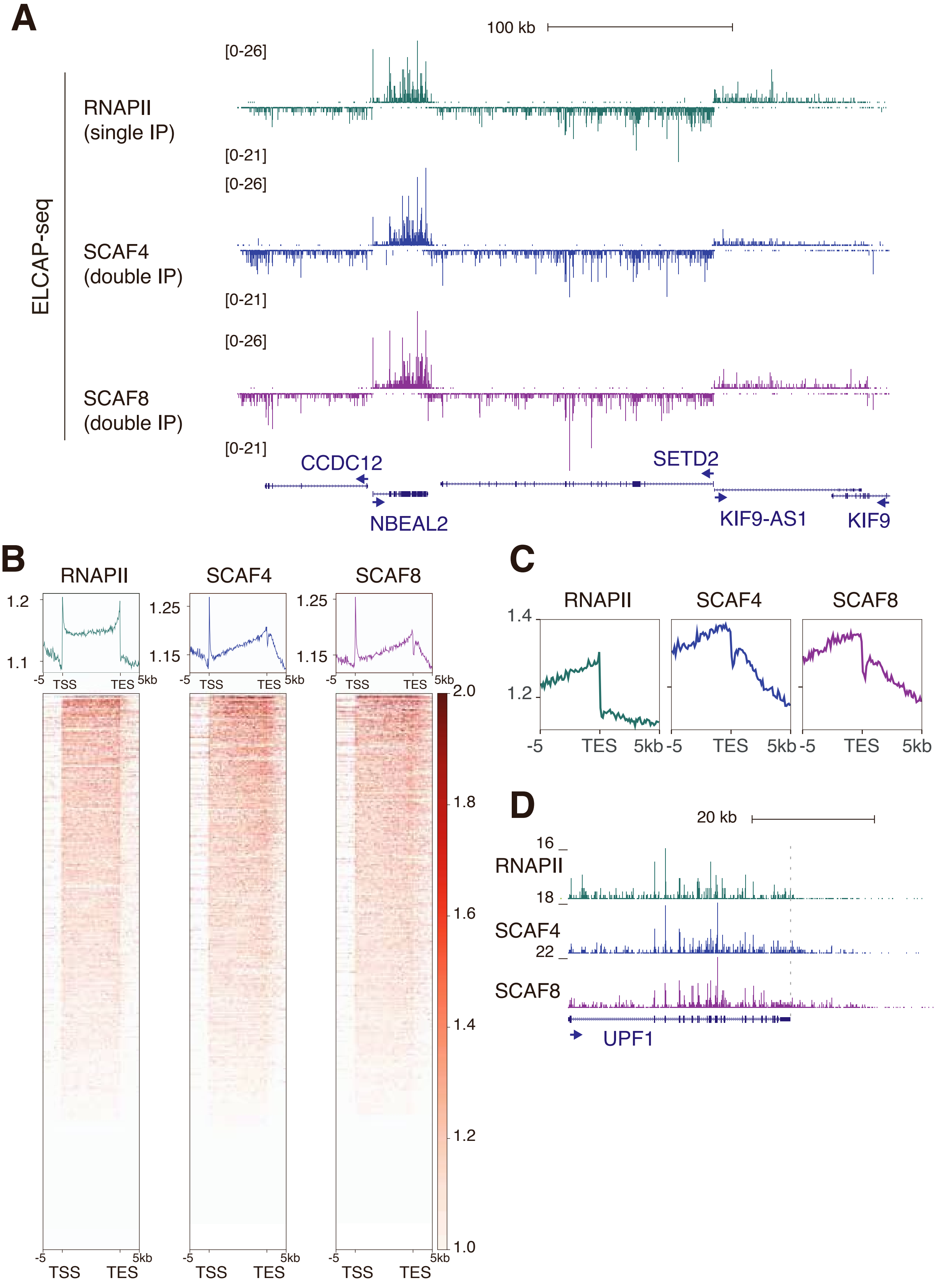

Supplemental Figure Legends

Supplemental Fig. 1. Establishment of ELCAP.

(**A**) Outline of single immunoprecipitation step of either RNAPII or FLAG-SCAF4. (**B**) Coomassie stain and Western blot of a single-step IP RNAPII purification for proteomics. Mock (IgG) IP was performed as a negative control (**C**) Correlation of log2 fold change enrichment between the two RNAPII ELCAP-MS (single IP) biological replicates.

Supplemental Fig. 2. Comparison of RNAPII ELCAP-MS and RNAPII mNET-MS.

(**A**) RNAPII ELCAP-MS (single step IP) log2 fold changes compared to RNAPII mNET-seq mass-spectrometry results for RNAPII complexes immunoprecipitated using a RNAPII CTD Ser2P antibody[1]. (**B**) As in A, but compared to RNAPII mNET-MS results from immunoprecipitation using a RNAPII CTD Ser5P antibody. (**C**) Venn diagram of RNAPII interactors identified with a log2 fold change >2 using RNAPII ELCAP-MS, RNAPII Ser2P mNET-MS and RNAPII Ser5P mNET-seq. (**D**) Heatmap showing 54 proteins with a log2 FC >2 using RNAPII ELCAP-MS, RNAPII Ser2P mNET-MS and RNAPII Ser5P mNET-seq. mNET-seq MS data is previously desribed[1].

Supplemental Fig. 3. Single and double ELCAP purification of RNAPII, SCAF4- and SCAF8-RNAPII.

(**A**) Single IP of FLAG-SCAF4 or RNAPII from stable FLAG-SCAF4 expressing cell lines. A FLAG IP or mock (IgG coupled to dynabeads) from HEK293 cells were used as negative controls (right part of gels). (**B**) Double affinity purification of SCAF4-RNAPII carried out by an initial FLAG IP, FLAG peptide elution and a subsequent RNAPII IP. A double affinity purification from HEK293 cells not expressing any FLAG-tag was used as a negative control (left side of gels). (**C**) Small RNA bioanalyzer chip result of RNA extracted from a two-step FLAG-RNAPII IP either from HEK293 cell (negative control) or from FLAG-SCAF4 cells. (**D**) Amount of RNA purified from RNAPII IP (single IP), SCAF4-RNAPII (double IP) and SCAF8-RNAPII (double IP), respectively. RNA concentrations were

measured by Qubit HS RNA assay. Data is shown for 3 biological replicates. For each sample a starting material of 15 x 15 cm dishes were used.

Supplemental Fig. 4. RNAPII ELCAP-seq profiles compared to ChIP-seq data.

Comparison of RNAPII ChIP-seq[2] with RNAPII ELCAP-seq (single IP). Both IPs are performed with the same antibody (4H8 monoclonal) against the phosphorylated RNAPII CTD. Data is shown both at the read level (top) and single nucleotide resolution (bottom). Zoom-in below highlights the RNAPII ChIP-seq and RNAPII ELCAP-seq (single IP) data for the ALDH7A1 gene.

Supplemental Fig. 5. Transcriptionally engaged RNAPII, SCAF4-RNAPII and SCAF8-RNAPII ELCAP-seq profiles.

(**A**) ELCAP-seq unnormalized genome browser track of single nucleotide resolution data for transcriptionally engaged RNAPII (single IP), SCAF4-RNAPII (double IP), SCAF8-RNAPII (double IP). (**B**) Density scaled metagene profiles (top) and heatmaps of transcriptionally engaged RNAPII, SCAF4-RNAPII and SCAF8-RNAPII across protein coding genes (n = 19,919). (**C**) ELCAP-seq coverage of transcriptionally engaged RNAPII (single IP), SCAF4-RNAPII (double IP) and SCAF8-RNAPII (double IP) centred around the transcript end site (TES) for all protein coding genes (n = 19,919). (**D**) ELCAP-seq for RNAPII (single IP), SCAF4-RNAPII (double IP) and SCAF8-RNAPII (double IP) mapping to the UPF1 gene.

Supplemental References

1. Nojima, T., Rebelo, K., Gomes, T., Grosso, A.R., Proudfoot, N.J., and Carmo-Fonseca, M. (2018). RNA Polymerase II Phosphorylated on CTD Serine 5 Interacts with the Spliceosome during Co-transcriptional Splicing. Mol Cell *72*, 369-379 e364. 10.1016/j.molcel.2018.09.004.
2. Zatreanu, D., Han, Z., Mitter, R., Tumini, E., Williams, H., Gregersen, L., Dirac-Svejstrup, A.B., Roma, S., Stewart, A., Aguilera, A., and Svejstrup, J.Q. (2019). Elongation Factor TFIIS Prevents Transcription Stress and R-Loop Accumulation to Maintain Genome Stability. Mol Cell *76*, 57-69 e59. 10.1016/j.molcel.2019.07.037.

Methods S1, related to STAR Methods

ELCAP for sequencing and mass-spectrometry: Step-by-step protocol

Cellular fractionation

1. Grow 2-5 x15cm dishes per condition. If using Flp-In T-REx HEK293 stably expressing Dox-inducible FLAG-tagged proteins induce expression overnight by the addition of 1 µg/mL doxycycline.
2. Harvested cells by scraping in 2 mL ice-cold PBS per 15 cm dish. Pellet cells by centrifugation at 1,500 rpm for 5 min at 4 ºC.
3. Wash pellet once in ice-cold PBS. Either snap freeze cell pellet in liquid nitrogen and store at -80 ºC or proceed to cellular fractionation.
4. Resuspend cell pellet in 2 pellet volumes of hypotonic buffer (10 mM HEPES pH 7.5, 10 mM KCl, 1.5 mM $MgCl_2$, 20 mM NEM (E3876, N-ethylmaleimide, Merck), incubated on ice for 15 min and dounce homogenize using 20 strokes with a loose pestle at end of incubation.
5. Pellet nuclei by centrifugation at 3,900 rpm for 15 min at 4 ºC and collected supernatant as cytoplasmic fraction.
6. Resuspend the remaining pellet in 2 pellet volumes (original cell pellet volume) nucleoplasmic extraction buffer (20 mM HEPES pH 7.9, 1.5 mM $MgCl_2$, 150 mM potassium acetate, 10 % (v/v) glycerol and 0.05 % (v/v) NP-40), incubated on ice for 20 min.
7. Centrifuge at 20,000g for 20 min at 4 ºC and remove supernatant as nucleoplasmic fraction. *Optional: This can be pooled with cytoplasmic fraction to obtain a combined soluble fraction.*
8. Resuspend the remaining pellet in chromatin digestion buffer (20 mM HEPES pH 7.9, 1.5 mM $MgCl_2$, 10 % (v/v) glycerol, 150 mM NaCl, 0.1 % (v/v) NP-40 and 250 U/mL Benzonase (Merck Millipore, 70746-4)) and incubate rotating for 1 hour at 4 ºC.

9. Centrifuge Benzonase digested samples at 20,000g for 20 min at 4 ^{o}C and collect supernatant as chromatin fraction.

Immunoprecipitations

1. For each sample (double IP) consisting of approximately 3 mL of chromatin extract, wash 100 uL anti-FLAG M2 Affinity Gel (A2220, Merck) three times in ice-cold PBS containing 0.05% NP-40. After the final wash resuspend in twice the original bead volume.
2. If chromatin extracts have been frozen prior to IP, re-clear extracts by 20 min centrifugation at 20,000g at 4 ^{o}C and collect supernatant as chromatin fraction for IPs.
3. Add 200 uL bead slurry (washed FLAG beads) to 3 mL of chromatin extracts and incubate at 4 ^{o}C for 1.5 h on a rotating wheel.
4. Wash bead 4 times with 5 mL IP wash buffer (150 mM NaCl, 20 mM Tris-HCl pH 7.5, 1.5 mM $MgCl_2$, 3 mM EDTA, 10 % (v/v) glycerol, 0.1 % (v/v) NP-40, phosphatase inhibitors (PhosSTOP, 04906837001, Merck) and protease inhibitor cocktail, 05056489001, Merck)). Transfer beads to a 15 mL falcon tube if not already in a falcon tube, centrifuge at 700 g for 5 min, remove supernatant without touching the pelleted beads. After the final wash resuspend in 200-500uL and transfer to a spin column (Thermo Fisher Scientific, 69705) placed in a 2 mL tube.
5. Wash twice on a spin column (Thermo Fisher Scientific, 69705). Add 200 uL wash buffer per wash, centrifuge at 700 g for 2 min at 4 ^{o}C and discard flow-through.
6. After the final wash, add a stopper (supplied with spin columns) to the bottom of the spin column followed by addition of 300 uL 1 mg/mL 3xFLAG peptide dissolved in IP wash buffer.
7. Incubate beads with FLAG-peptide elution buffer for 1 hour at 4 ^{o}C on a rotating wheel.
8. To confirm immunoprecipitation of FLAG-tagged proteins for the first IP step, save 5% of the FLAG elutions for later to run on an SDS-PAGE for western blot.

9. Dilute the remaining FLAG elution to 1mL per sample by addition of IP wash buffer and use for the subsequent RNAPII immunoprecipitation.
10. For each sample wash 50 uL Dynabeads Protein G (10004D, Thermo) 3 times in PBS, 0.05% NP-40 and incubated with 5 ug of RNAPII 4H8 antibody in a total volume of 1.2 mL for 2 hrs at room temperature.
11. Wash 4H8-conjugated beads 3 times in PBS, 0.05% NP-40, resuspended in 100 uL PBS, 0.05% NP-40 and added to the FLAG elution for the first IP step.
12. Incubate samples 2 hrs at 4 ^{o}C on a rotating wheel.
13. Wash beads on a magnetic stand 5 times in IP wash buffer.
14. To confirm double IP of RNAPII, save 5% of the beads removed for a western blot control of the immunoprecipitation.
15. Proceed with remaining bead pellet for ELCAP-seq or ELCAP-MS as described below.

Preparation of RNA for sequencing (ELCAP-seq)

1. Use the remaining beads from the double IP directly for RNA extraction by addition of 300 uL RNA extraction mix: consisting of 100 uL IP wash buffer + 100 uL Zymo RNA lysis buffer (R1050, Zymo Research Quick-RNA MicroPrep) + 100 uL 100% ethanol) directly to the dry beads.
2. Incubated beads 2 min with the RNA extraction mix at room temperature and place back on the magnetic stand.
3. Transfer supernatant containing the RNA to a new tube and use for isolation of both small (17-200nt RNA) and >200nt RNA using the Zymo Research Quick-RNA MicroPrep (R1050) accordingly to the manufacturer's instructions. Elute purified RNA in 15 uL RNase-free water.
4. To assess size distribution of purified RNA, run 1-3 uL on a small RNA bioanalyzer chip (2100 Bioanalyzer Agilent).
5. Measure RNA concentration by Qubit/RNA HS assay (Q32852, Thermo) accordingly to the manufacturer's instructions.

6. Use 5 uL of isolated RNA for sequencing library prep using the NEBNext Multiplex Small RNA Library Prep kit for Illumina (E7300, NEB). There is no need for end-repair of the RNA prior to the library prep as the Benzonase generated ends are compatible with adapter ligations.
7. Amplify libraries using 9-12 PCR cycles (accordingly to input material, consult the NEBNext Multiplex Small RNA Library Prep manual).
8. Run PCR products a 6 % Novex TBE gel (EC6265BOX, Thermo). Stain gel with SYBR Gold (S11494, Thermo) for 10 min and cut out product within the size range of 140-230 bp (corresponding to an insert size range of 20-90 nt).
9. Crush Gel slices with a RNase-free single-use pestle (12-141-364, Fisher Scientific) and incubated in 250 uL gel elution buffer (supplied with NEBNext kit) for 2 hrs at room temperature.
10. Transfer gel pieces to a Spin-X gel filtration column (CLS8160, Merck) and centrifuged for 2 min at 13.000 rpm.
11. Collect flow-through and precipitate DNA by addition of 750 uL 100 % ethanol, 25 uL 3 M sodium acetate pH 5.5 and 1 uL linear acrylamide (supplied with NEBNext kit).
12. Incubate overnight at -20 ºC.
13. The following day, centrifuge samples at 13.000 rpm for 30 min at 4 ºC.
14. Wash pellets in 80% ethanol (make fresh), air-dry and resuspend in 10 uL TE buffer.
15. Measure DNA concentration of the PCR library by Qubit/HS dsDNA kit (Q32851, Thermo).
16. Preform library QC to confirm size range on an Agilent 4200 TapeStation.
17. Sequence samples on a HiSeq4000 (Illumina) (SE75 run) or similar.

Proteomics of RNAPII complexes (ELCAP-MS)

1. Use the remaining beads from the single or double IP step above to eluate bound proteins: Add 50 uL glycine elution buffer (100 mM glycine pH 2.4) directly to dry beads, incubate 5 min at room temperature and vortex.

2. Transfer supernatant containing eluted proteins to a new tube and neutralise by addition of 25 uL 1 M Tris pH 8.8.
3. Add an equal volume of 2x SDS containing loading buffer run samples on SDS-PAGE.
4. Run gel at 100 V for approximately 10 min to allow samples to migrate 2 cm into the gel. Cut out entire gel piece containing stained proteins.
5. Preform in-gel digested with trypsin, using a Janus Automated Workstation (Perkin Elmer).
6. Analyze peptides using an LTQ Orbitrap-Velos mass spectrometer coupled to an Ultimate3000 HPLC equipped with an EASY-Spray nanosource (Thermo Fisher Scientific) or similar setup.

ELCAP-seq analysis

1. Trim reds using TrimGalore v0.4.4 (Martin, 2011). Discard reads < 10 bp in length and those with a maximum error rate > 0.05.
2. Align remaining reads using HISAT2 v2.0.4 against the GRCh38 genome build in a strand-specific manner, allowing for at most 5 distinct primary alignments for each read (Kim et al., 2015).
3. Sort and index reads using SAMtools (Handsaker et al., 2009). Remove multi-mapping reads.
4. Remove duplicate reads using Picard.
5. Create bedgraph files directly from the filtered single-end BAM files using BEDTools (Quinlan et al., 2010) and thereafter bigwig files using bedGraphToBigWig (Kent et al., 2010). Create bigwig files at maximum (i.e., single-bp, not binned) resolution.
6. For feature profiles (metagene plots, TSS and pA centred plots), density scale data after bin size selection to allow comparison of binding behaviour between RNAPII reference (single IP) and the RNAPII subpopulation (double IP).

Troubleshooting tips

Issue	Solution
No FLAG-IP	Check expression of FLAG-tagged protein in input samples using WB. For dox-inducible cell lines make sure dox induction is carried out for a least 14 hrs.<br><br>If FLAG protein is expressed in input samples but fails to IP, try tagging the protein of interest at other terminus or adding a linker between the tag and protein.
No RNAPII IP	Make sure 4H8 is conjugated to beads.<br><br>Run a silver stain with 1/100 dilution input sample, 1/100 dilution of unbound and a few uL of the IP sample.
Both RNAPII single IP and FLAG single IP works, but not double FLAg-RNAPII IP	Check that your factor of interest is associated with elongating RNAPII e.i. IPed by the anti-4H8 antibody. See Table S1.<br><br>If your factor of interest interacts exclusively with unphosphorylated RNAPII, consider using the 8WG16 antibody for the single RNAPII refence IP instead.
No RNA co-IPed	Take care to work RNase-free during the RNA extraction as small RNA fragments are no longer protected from degradation by the RNAPII complex.<br><br>Make sure that the double FLAG-RNAPII IP works and use the single RNAPII 4H8 IP as a positive control compared to a double FLAG-RNAPII-4H8 IP from cells not expressing a FLAG-tagged protein as a negative control.
RNA from single RNAPII IP samples, but not in factor-specific IP	Make sure that the single FLAG IP is efficient.<br><br>If background is an issue, shorten the incubation with the FLAG beads and scale down the amount of FLAG beads/extract volume.<br><br>If the control is clean, but the FLAG-IP yield for the factor of interest is low consider scaling up the amount of input material. Make sure expression of FLAG-tagged protein (see point above) is alright.
RNA sizes > 100 nt in IP	Make sure Benzonase enzyme has been stored correctly. Keep aliquots and avoid extended time outside freezer.<br><br>Increase incubation time with Benzonase to ensure complete digestion of unprotected DNA/RNA.
Appearance of adapter-adapter ligations peak after gel purification	Use a lower dilution of the RNA adapter (1:5 or 1:10) for low RNA input samples during the library prep.

	If issues persist, consider introducing a AMPure bead purification prior to the TBE gel purification.
The DNA library sizes appears as a smear on the TBE gel	The appearance of large fragment sizes can be due to overamplification of the library. Decrease the number of PCR cycles and if possible, test the number of optimal cycles from a test IP sample removing samples at cycle 6, 8, 10, and 12.
